# Cellular Calcium Levels Influenced by NCA-2 Impact Circadian Period Determination in *Neurospora*

**DOI:** 10.1128/mBio.01493-21

**Published:** 2021-06-29

**Authors:** Bin Wang, Xiaoying Zhou, Scott A. Gerber, Jennifer J. Loros, Jay C. Dunlap

**Affiliations:** a Department of Molecular and Systems Biology, Geisel School of Medicine at Dartmouth, Hanover, New Hampshire, USA; b Norris Cotton Cancer Center, Geisel School of Medicine, Dartmouth, Hanover, New Hampshire, USA; c Department of Biochemistry and Cell Biology, Geisel School of Medicine at Dartmouth, Hanover, New Hampshire, USA; Karlsruhe Institute of Technology (KIT)

**Keywords:** *nca-2*, calcium, FRQ, FRQ phosphorylation, Ca^2+^-CaM-dependent kinases, CAMK

## Abstract

Intracellular calcium signaling has been implicated in the control of a variety of circadian processes in animals and plants, but its role in microbial clocks has remained largely cryptic. To examine the role of intracellular Ca^2+^ in the *Neurospora* clock, we screened mutants with knockouts of calcium transporter genes and identified a gene encoding a calcium exporter, *nca-2*, uniquely as having significant period effects. The loss of NCA-2 results in an increase in the cytosolic calcium level, and this leads to hyper-phosphorylation of core clock components, FRQ and WC-1, and a short period, as measured by both the core oscillator and the overt clock. Genetic analyses showed that mutations in certain *frq* phospho-sites and in *Ca^2+^-calmodulin-dependent kinase 2* (*camk-2*) are epistatic to *nca-2* in controlling the pace of the oscillator. These data are consistent with a model in which elevated intracellular Ca^2+^ leads to the increased activity of CAMK-2, leading to enhanced FRQ phosphorylation, accelerated closure of the circadian feedback loop, and a shortened circadian period length. At a mechanistic level, some CAMKs undergo more auto-phosphorylations in the *Δnca-2* mutant, consistent with high calcium levels in the *Δnca-2* mutant influencing the enzymatic activities of CAMKs. NCA-2 interacts with multiple proteins, including CSP-6, a protein known to be required for circadian output. Most importantly, the expression of *nca-*2 is circadian clock-controlled at both the transcriptional and translational levels, and this in combination with the period effects seen in strains lacking NCA-2 firmly places calcium signaling within the larger circadian system, where it acts as both an input to and an output from the core clock.

## INTRODUCTION

In most eukaryotes and certain prokaryotes, circadian clocks link environmental cues, such as temperature and light, to metabolism to regulate various physiological and molecular events, ranging from virulence and immunity to cell cycle control (1–3). In fungi and mammals, the core circadian machinery is built based on a transcriptional-translational feedback mechanism in which the positive arm drives the transcription of components comprising the negative arm, which, in turn, feeds back to repress the positive arm, terminating its own expression. Neurospora crassa has been widely used as a model eukaryote for circadian studies for decades. In *Neurospora*, the White Collar complex (WCC), formed from WC-1 and WC-2, serves as the positive-arm transcriptional activator for the core clock gene *frequency* (*frq*) by binding to one of two DNA elements, the *Clock box* (*C-box*) in the dark or the *Proximal Light-Response Element* (*PLRE*) in the light (4–6). FRQ, the gene product of *frq*, interacts with FRH (FRQ-interacting RNA helicase) (7, 8) and casein kinase I (CKI) (9) to form the FFC complex, the negative arm that represses WCC activity by promoting its phosphorylation at a group of residues (10).

Protein phosphorylation has been shown to control protein functions via protein-protein/DNA associations, protein stability and activity, and subcellular localization, all of which have been proven or suggested to regulate functions of circadian components (11–14). In *Neurospora*, FRQ is intricately regulated by over 100 time-specific phosphorylation events (9, 15); multiple kinases, such as CKI, CKII, protein kinase A (PKA), and Ca^2+^-calmodulin (CaM)-dependent kinase 1 (CAMK-1), and phosphatases, like PP2A, have been reported to directly or indirectly control FRQ phosphorylation status (16–18). Extensive phosphorylation has also been observed on WCC under light and dark conditions (10, 16, 19, 20). Recently, over 90 phosphoresidues have been mapped on WC-1 and WC-2, governing their circadian repression and controlling circadian output, and a small subset of these has been shown to be essential for feedback loop closure (10).

Calcium as a second messenger regulates a wide variety of cellular pathways. For example, elevated Ca^2+^ in the cytosol and mitochondria of neurons is required to synchronize neuronal electrical activity (e.g., reviewed in reference 21), all muscle fibers use Ca^2+^ as their main regulatory and signaling molecule (e.g., reviewed in reference 22), and Ca^2+^ influx induces oocyte development in many species during mammalian fertilization (23). At the molecular level, enzymes and other proteins can be regulated by calcium via allosteric regulatory effects (24). Indeed, diverse evidence also connects calcium signaling with circadian regulation. In Arabidopsis thaliana, the concentration of cytosolic Ca^2+^ oscillates over time (25, 26), which regulates circadian period length through the action of a CALMODULIN-LIKE protein on the core circadian oscillator (27). Circadian oscillation of Ca^2+^ has been observed in hypothalamic suprachiasmatic nucleus (SCN) neurons, driving daily physiological events (28). In addition, a small body of literature has described effects of calcium ionophores and calmodulin antagonists on the *Neurospora* clock (29–33). Although this research was published before there was sufficient understanding of basic cellular physiology to fully interpret the work, it provides a rich context for studies on the role of calcium signaling in the *Neurospora* clock.

Despite the paucity of recent data on circadian effects of calcium in fungi, the cellular physiology of calcium metabolism in fungi, including *Neurospora*, is well understood (34–40) and is consistent with general knowledge of animal cells. The resting concentration of Ca^2+^ in the cytoplasm of fungal and mammalian cells is normally maintained at 50 to 200 nM (41–45), which is 20,000- to 100,000-fold lower than that in a typical extracellular environment (46). To be maintained at this low level in the cell, Ca^2+^ is actively pumped out from the cytosol to the extracellular space, reticulum, vacuole, and/or mitochondria (34, 35, 47–51); bearing binding affinity to Ca^2+^, certain proteins in the cell can also contribute to lowering the level of free cytosolic Ca^2+^ (52).

To elicit signaling events, the cell releases Ca^2+^ from organelles or Ca^2+^ enters the cell from extracellular environments. When stimulated by certain signals, cytoplasmic Ca^2+^ can be suddenly increased to reach ∼500 to 1,000 nM through activation of certain ion channels in the endoplasmic reticulum (ER) and plasma membrane or indirect signal transduction pathways, such as G protein-coupled receptors (e.g., reviewed in references 53 and 54). Cytosolic calcium bursts lead to activation of CAMKs (55–59). In mammals, the CAMK cascade includes three kinases: CaM kinase kinase (CaMKK), CaMKI, and CaMKIV. CaMKI and CaMKIV are phosphorylated and activated by CaMKK (55, 60–65). CaMKK and CaMKIV reside in the nucleus and cytoplasm, while CaMKI is located only in the cytosol. Nuclear CaMKIV promotes the phosphorylation of several transcription factors, such as CREB and CBP, to regulate gene expression (60, 66, 67). The *Neurospora* genome encodes four CAMK genes that are subject to diverse regulation, although little is known about their intracellular localization (18, 37).

By impacting a wide range of cellular processes, circadian clocks and calcium signaling are two classic regulatory mechanisms evolved to coordinate environmental factors, cellular responses, and metabolism. In this study, a screen of calcium regulators identified *nca-2*, a calcium pump gene, as a regulator of circadian period length in *Neurospora.* In Δ*nca-2* strains, FRQ and WC-1 become hyper-phosphorylated; deletion of *camk-2* individually blocks the period-shortening effect and FRQ hyper-phosphorylations in the Δ*nca-2* mutant. NCA-2 interacts with multiple proteins, which suggests that it might function in cellular processes in addition to the circadian clock.

## RESULTS

### Identification of *nca-2* as a regulator of the *Neurospora* circadian clock.

**FIG 1:**
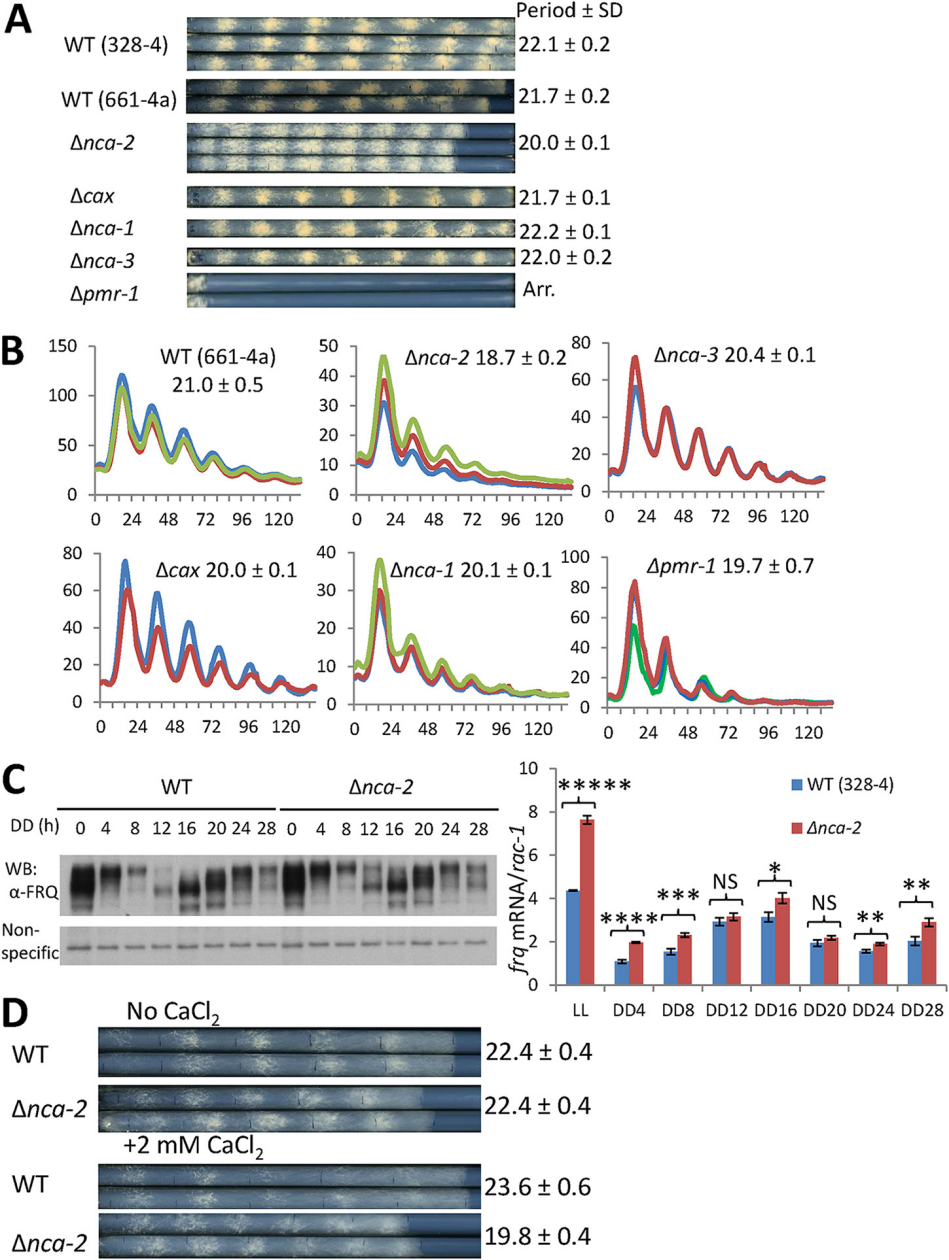
Gene deletions of calcium pumps were tested for circadian phenotypes by race tube (A) and luciferase (B) analyses. Strains were cultured on 0.1% glucose race tube medium in a 96-well plate and synchronized by growth in constant light overnight (16 to 24 h), followed by transfer to darkness. Bioluminescence signals were monitored with a CCD camera every hour, bioluminescence data were acquired using ImageJ with a custom macro, and period lengths were manually calculated. Raw bioluminescence data from three replicates were plotted with the *x* axis and *y* axis representing time (in hours) and arbitrary units, respectively. (C, left) Western blot showing the expression level of FRQ in the WT and the Δ*nca-2* mutant over 28 h detected with FRQ-specific antibody (α-FRQ). DD, number of hours after the light-to-dark transfer. (right) RT-qPCR showing relative levels of *frq* mRNA expressed in the WT and the Δ*nca-2* mutant. *rac-1* was used as an internal control, to which *frq* expression is normalized (*n* = 3, mean values ± standard errors of the means). Asterisks indicate statistical significance in a comparison with the WT as determined by a two-tailed Student *t* test. *****, *P* < 0.00001; ****, *P* = 0.00006; ***, *P* = 0.001337; **, *P* < 0.01; *, *P* = 0.010131; NS, the difference is not significant. (D) Race tube assays of the WT and the Δ*nca-2* mutant strain using race tube media in the presence or absence of 2 mM calcium chloride. Growth fronts of the strains were marked by vertical black lines every 24 h. *nca-3* (NCU05154), the calcium P-type ATPase; *nca-1* (NCU03305), the calcium-transporting ATPase sarcoplasmic/endoplasmic reticulum type; *cax* (NCU07075), the calcium/proton exchanger; *pmr-1* (NCU03292), the calcium-transporting ATPase type 2C member 1; *nca-2* (NCU04736), the plasma membrane calcium-transporting ATPase 3. Gene names, numbers beginning with “NCU,” and descriptions were obtained from the FungiDB website (https://fungidb.org/fungidb/app). The period was determined as described in Materials and Methods and is reported ± standard deviations (SD) (*n* = 3).

Calcium signaling impacts circadian processes (see, e.g., references 18, 30, and 31) and directly controls a wide range of cellular and physiological events, but the means through which it impacts the circadian system is not fully described. *Neurospora* encodes several calcium transporter genes, including *nca-1* (a sarco/endoplasmic reticulum Ca^2+^-ATPase [SERCA]-type ATPase), two closely related genes, *nca-2* and *nca-3* (plasma membrane Ca^2+^-ATPase [PMCA]-type ATPases), *pmr-1* (a secretory pathway Ca^2+^-ATPase [SPCA]-type Ca^2+^ ATPase), and *cax* (a vacuolar Ca^2+^/H^+^ exchanger) (35). To facilitate monitoring of circadian phenotypes, individual strains with these calcium pump genes knocked out were backcrossed to *ras-1^bd^* and *frq C-box*-driven *luciferase* strains and analyzed by race tube and luciferase assays. Of these deletion mutants tested, the Δ*pmr-1* mutant shows an extremely slow growth rate on race tubes (Fig. 1A) but is nicely rhythmic, with a slightly shorter period, in the luciferase assay (Fig. 1B); disruption of *nca-2*, a plasma membrane-located calcium pump, leads to an ∼2-h-shorter period than that of the wild type (WT) by race tube (Fig. 1A) and luciferase (Fig. 1B) analyses. (Of note, although on any given day the period estimates of strains bearing mutated calcium pumps showed normal precision, period length assays done on different days were more varied than is typical. For this reason, comparisons within figures always reflect assays of different strains done on the same day with the same medium.) Appearing after 12 hours in constant-darkness (DD12), newly synthesized FRQ in the Δ*nca-2* mutant is slightly more abundant than in the WT (Fig. 1C, left) and *frq* mRNA levels in the subjective circadian night phase (DD4, -8, -24, -28) of the *Δnca-2* mutant are substantially higher than in the WT (Fig. 1C, right), consistent with a faster-running circadian clock in the Δ*nca-2* mutant (Fig. 1A and B). The cytosolic calcium level in the Δ*nca-2* mutant is increased about 9.3-fold compared to that in the WT (36), suggesting a basis for this period change. To verify that the period shortening in the Δ*nca-2* mutant was due to this increased intracellular Ca^2+^, the *Δnca-2* strain was examined on race tubes prepared without calcium in the medium. Interestingly, in Ca^2+^-free medium, the Δ*nca-2* mutant displays a WT period on race tubes, while with normal levels of calcium in the medium, its clock becomes ∼4-h shorter than that of the WT (Fig. 1D), confirming that the role of *nca-2* in regulating the pace of the circadian oscillator is through controlling the cytosolic calcium level. These data indicate that *nca-2* is required for keeping calcium in the cytosol at reduced levels to maintain a normal circadian period.

### WC-1 and FRQ are hyper-phosphorylated in the *Δnca-2* mutant.

**FIG 2:**
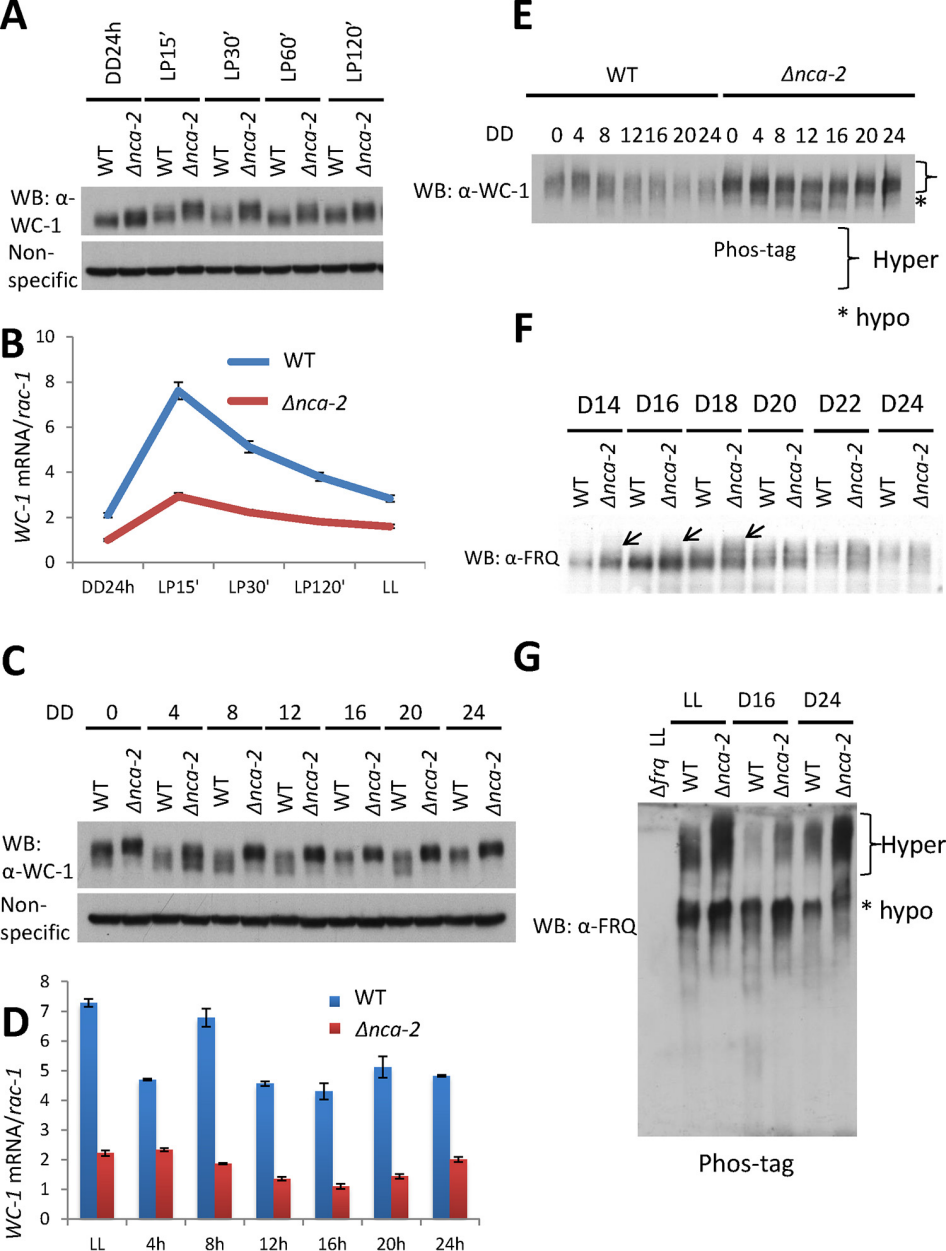
The circadian components WC-1 and FRQ are hyper-phosphorylated in the Δ*nca-2* mutant. (A) Total WC-1 was monitored by Western blotting. Samples were cultured in constant darkness prior to a 15-, 30-, 60-, and 120-min light exposure. Nonspecific bands in the same blot are shown for equal loadings. Decreased electrophoretic mobility is indicative of phosphorylation status (7). (B) mRNAs extracted from samples cultured in the dark for 24 h or following a 15-, 30-, or 120-min light exposure, as indicated, were reverse transcribed to cDNA, followed by quantitative PCR with a primer set specific to *wc-1*. (C) Western blotting of WC-1 in a 24-h time course with a 4-h interval. (D) As in panel C, RT-qPCR was performed with samples harvested under the circadian conditions indicated. Phosphorylation profiles of WC-1 (E) and FRQ (F, G) in the WT and the Δ*nca-2* mutant were analyzed by Western blotting using SDS-PAGE gels bearing 20 μM Phos tag chemicals and a ratio of 149:1 acrylamide to bisacrylamide (G). (F) Western blotting of FRQ in the WT and the Δ*nca-2* mutant from DD14 to DD24 with a 2-h resolution. * in panel E denotes the mobility of unphosphorylated WC-1 and the bracket the region corresponding to hyper-phosphorylated WC-1. Arrows indicate hyper-phosphorylated FRQs observed in the Δ*nca-2* mutant.

WC-1 and FRQ are essential components in the positive and negative arms, respectively, of the *Neurospora* feedback loop, and their phosphorylation has been proven to play an essential role in determining their circadian functions (9, 10, 15, 16, 19). In addition to serving as the main transcription factor driving the expression of *frq*, WC-1 is the principal blue light photoreceptor for the organism, forming a homodimer (4) and getting hyper-phosphorylated (20) upon light exposure. To probe WC-1 and FRQ in the Δ*nca-2* mutant, amounts and phosphorylation profiles of WC-1 and FRQ were analyzed by Western blotting using specific antibodies. The stability of FRQ in the Δ*nca-2* mutant is very similar to that in the WT (Fig. S1), and although WC-1 appeared slightly less stable, the cellular levels of WC-1 were even above those of the WT, altogether suggesting that the stability of the core clock components does not determine the shortened period in the Δ*nca-2* mutant and that WC-1’s level and stability are not consistent with the period length shortening in the Δ*nca-2* mutant. Following a light pulse, WC-1 is more abundant and hyper-phosphorylated in the Δ*nca-2* mutant than in the WT (Fig. 2A), whereas, surprisingly, expression of *wc-1* is significantly lower than that in the WT (Fig. 2B). Consistent with the data from the light pulse experiment, in the dark, the Δ*nca-2* mutant contains a higher level of WC-1 with more phosphorylations (Fig. 2C) despite a low mRNA level (∼20 to 50% of the level in the WT) (Fig. 2D). These data suggest that *nca-2* regulates *wc-1* expression at both the transcriptional and posttranscriptional levels independently of light and dark conditions. The hyper-phosphorylation of WC-1 in the Δ*nca-2* mutant was confirmed by a more sensitive assay (Fig. 2E) using Phos tag gels (68), such as have been applied to resolve single phosphoresidues on WC-1 and WC-2 (10). Like WC-1, FRQ in the Δ*nca-2* mutant is also more heavily phosphorylated than in the WT at DD14, -16, and -18 (Fig. 2F), when newly synthesized FRQ is the dominant form in the cell, and at DD24 (Fig. 2G), when all FRQ becomes extensively phosphorylated prior to its turnover (Fig. 1A). All together, these data demonstrate that WC-1 and FRQ become hyper-phosphorylated in the Δ*nca-2* mutant, suggesting that the elevated calcium in the Δ*nca-2* mutant might lead to an overactivation of a kinase(s) or repression of a phosphatase(s) targeting FRQ and WC-1, thereby altering their activities in the clock.

10.1128/mBio.01493-21.1 FIG S1 Stability of WC-1 and FRQ in the WT and the Δ*nca-2* mutant. (A, left) Cycloheximide (CHX) was added to the *Neurospora* culture growing in the light at a final concentration of 40 μg/ml, and tissues were harvested at the indicated time points and assayed by Western blotting with WC-1- and FRQ-specific antibodies. (Right) Densitometric analyses of the blots on the left showing WC-1 and FRQ levels in the WT and the Δ*nca-2* mutant treated with 40 μg/ml CHX for 0, 4, 8, or 12 h, as indicated. (B, left) FRQ stability in the WT and the Δ*nca-2* mutant is measured using CHX-treated cultures sampled every 2 h over 14 h. (Right) Densitometric analysis of FRQ in the left panel. Download FIG S1, PDF file, 0.1 MB. Copyright © 2021 Wang et al. 2021 Wang et al. https://creativecommons.org/licenses/by/4.0/ This content is distributed under the terms of the Creative Commons Attribution 4.0 International license.

### Epistasis analysis is consistent with an effect of the Δ*nca-2* mutant on FRQ but not on WCC.

**FIG 3:**
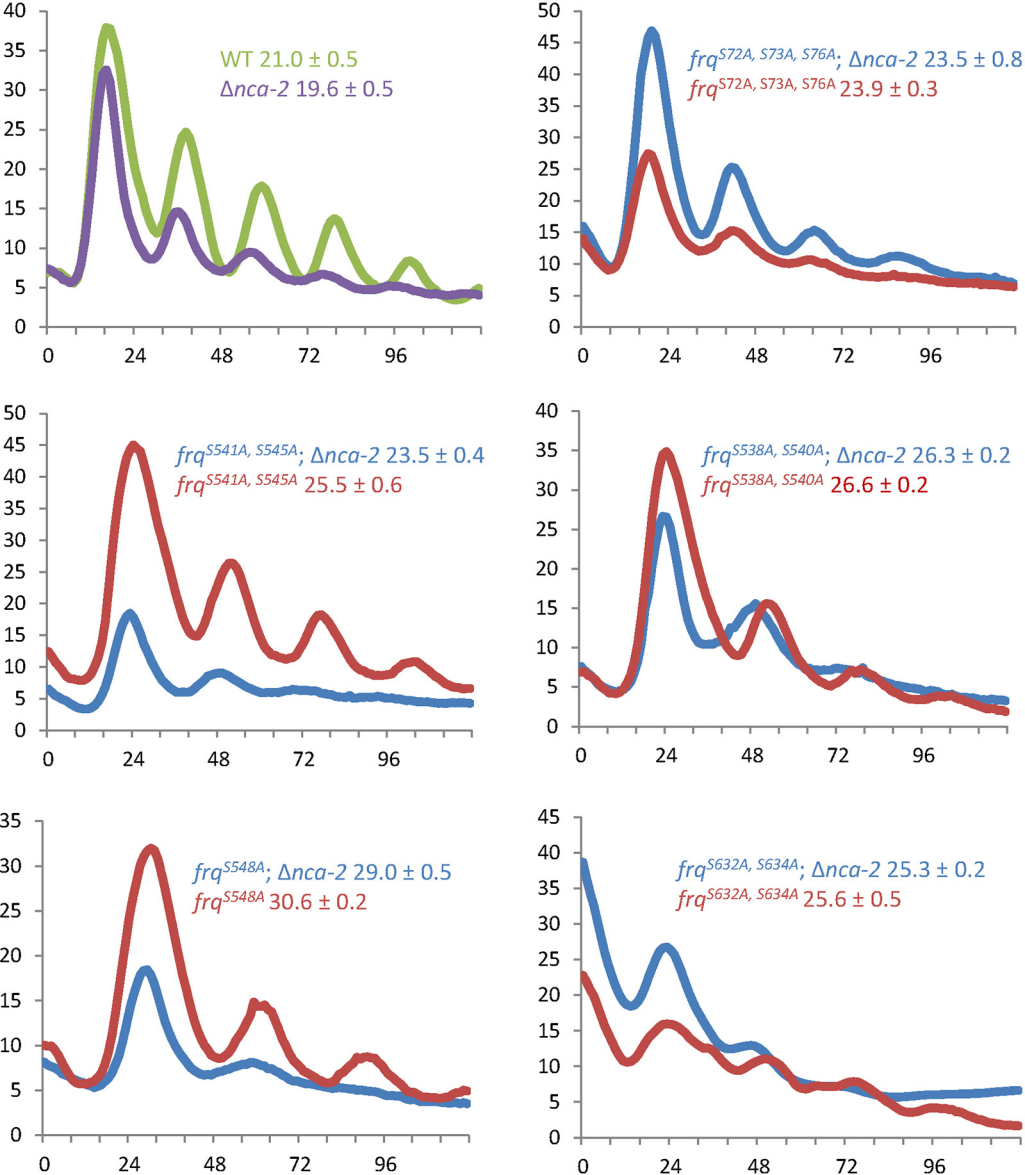
Some *frq* alleles are epistatic to the Δ*nca-2* mutant. The *frq C-box* promoter activity was measured using *C-box–luciferase* at the *his-3* locus in the indicated *frq* phospho-mutants in the presence or absence of *nca-2*. Strains were grown on 0.1% glucose race tube medium in constant light overnight (16 to 24 h) prior to transfer to darkness. The *frq^S72A^*^,^ *^S73A^*^,^ *^S76A^*, *frq^S541A^*^,^ *^S545A^*, *frq^S538A^*^,^ *^S540A^*, *frq^S548A^*, and *frq^S632A^*^, S634A^ mutants were derived from reference 9. Period was determined as described in Materials and Methods and is reported ± SD (*n* = 3).

FRQ is phosphorylated in a time-specific manner at over 100 sites, and elimination of certain phospho-sites in different domains can cause opposite phenotypes on period lengths (9, 15). Because the loss of *nca-2* elicits FRQ hyper-phosphorylation at almost all time points examined (Fig. 2F and G), we reasoned that this enhanced FRQ phosphorylation in the Δ*nca-2* mutant might contribute to the short period length in this strain. If this is so, then circadian period lengths in *frq* mutants encoding proteins that cannot be phosphorylated at key residues should not be shortened. To this end, several *frq* phospho-mutants displaying long circadian periods from reference 9 were individually backcrossed to Δ*nca-2* and *frq-luc* strains and assayed by tracking bioluminescent signals in real-time in darkness. While circadian periods of *frq^S541A^*^,^ *^S545A^*, *frq^S548A^*, and *frq^7^* mutants responded to a loss of *nca-2*, as did the WT (Fig. 3 and see Fig. S2A in the supplemental material), the absence of *nca-*2 does not significantly influence the period length of the *frq*^S72A,^ *^S73A^*^,^ *^S76A^*, *frq^S538A^*^,^ *^S540A^*, or *frq^S632A^*^,^ *^S634A^* mutants (Fig. 3). These proteins cannot be phosphorylated at these residues, which results in period lengthening (9), so the epistasis of these *frq* alleles is consistent with NCA-2 influencing FRQ phosphorylation at these sites.

10.1128/mBio.01493-21.2 FIG S2 The Δ*nca-2* mutant shortens the period length in a long-period *frq* allele and in *wcc* phospho-mutants. (A) The activity of the *frq* promoter is measured by *C-box–luc* bioluminescence in the background of *frq*^7^, *wc-1^S971A^*^,^ *^S988A^*^,^ *^S990A^*^,^ *^S992A^*^,^ *^S994A^*^,^ *^S995A^*, or *wc-2^S433A^* in the presence or absence of *nca-2*, as indicated. (B) Phosphorylations of WC-1 S971 and S990 in the presence or absence of *nca-2* were assayed using a 6.5% SDS-PAGE gel bearing Phos tag (for details, see Materials and Methods). The phosphorylation of WC-1 residues S971, S988, S990, S992, S994, and S995 is promoted by FRQ and essential for the closure of the circadian feedback loop (10). Download FIG S2, PDF file, 0.1 MB. Copyright © 2021 Wang et al. 2021 Wang et al. https://creativecommons.org/licenses/by/4.0/ This content is distributed under the terms of the Creative Commons Attribution 4.0 International license.

To examine the effect of *nca-2* deletion on WCC phosphorylation and period length in the same manner, the Δ*nca-2* mutant was backcrossed to several *wcc* mutants in which key phosphoresidues that have been identified and shown to determine the circadian feedback loop closure (10) were eliminated, and the strains were monitored by the luciferase assay. The absence of *nca-2* further shortens the periods of *wc-1^S971A^*^,^ *^S988A^*^,^ *^S990A^*^,^ *^S992A^*^,^ *^S994A^*^,^ *^S995A^* and *wc-2^S433A^* strains (Fig. S2A), suggesting that *nca-2* regulates the core oscillator independently of WCC phosphorylation at the sites essential for its repression. Consistently with this, in the Δ*nca-2* mutant, the phosphorylation levels of WC-1 S971 and S990, two key sites required for FFC-mediated WCC repression, are similar to that in the WT (Fig. S2B), further suggesting that altered phosphorylation of the positive arm in the oscillator is not the cause of the short period of the Δ*nca-2* mutant.

### *camk-2* deletion does not further shorten the period of the Δ*nca-2* mutant.

**FIG 4:**
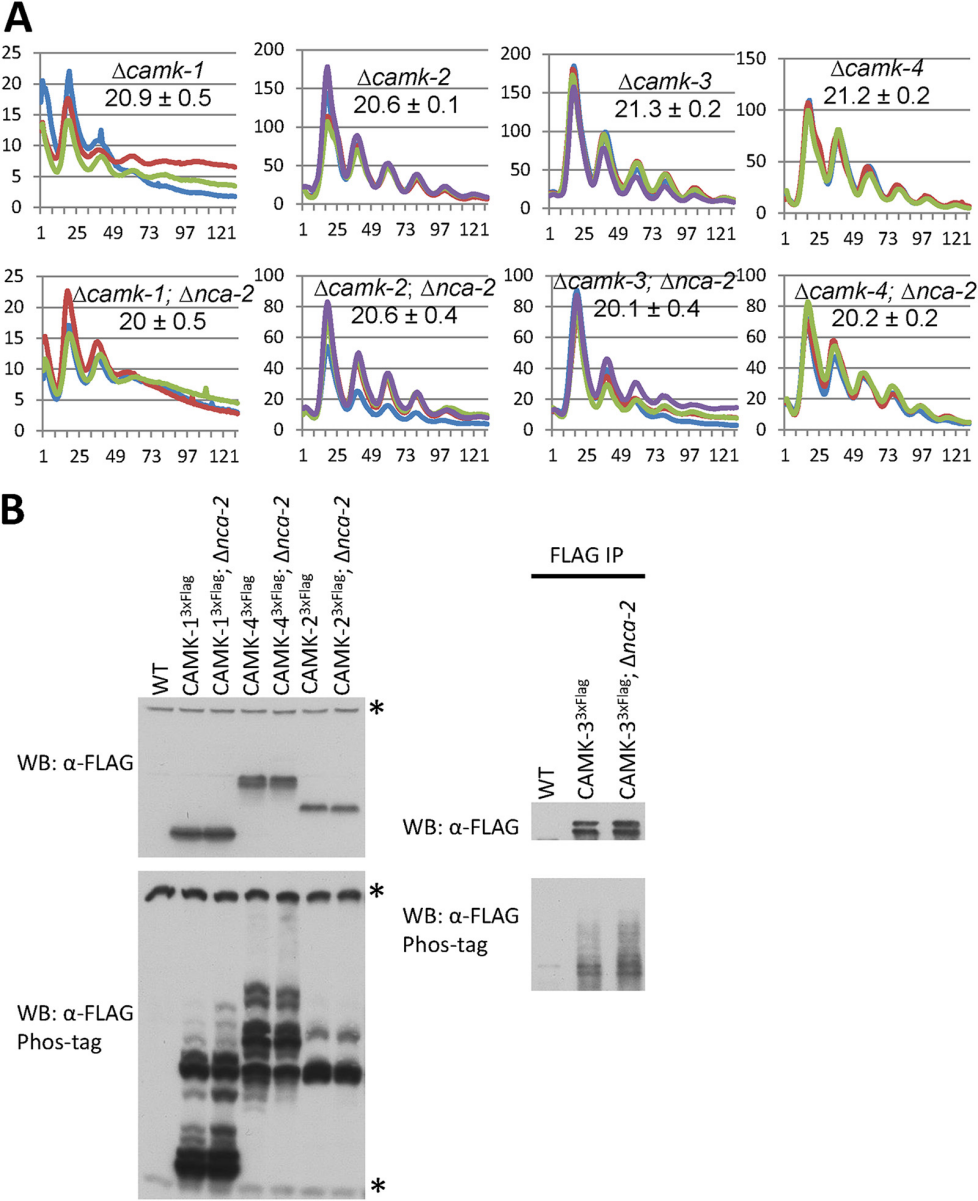
Period shortening of the Δ*nca-2* mutant is rescued by deletion of *camk-2*. (A) Luciferase assays were performed with a *frq C-box* promoter-driven *luciferase* gene at the *his-3* locus in individual *camk-1* to -*4* knockouts in the presence or absence of *nca-2*, as indicated. Periods (in hours) are reported as described in Materials and Methods and are reported ± SD (*n* = 3). (B, top) Total levels of CAMK-1 to -4, which have a 3×FLAG tag at their C termini, in the WT or the *Δnca-2* background were assayed by Western blotting with FLAG antibody. (Bottom) Phosphorylation profiles of CAMK-1 to -4 were analyzed for the same sample set with 149:1 acrylamide to bisacrylamide SDS-PAGE gels containing the Phos tag. Asterisks indicate nonspecific bands. For CAMK-1, -2, and -4, total lysates were applied, while CAMK-3 was first pulled down by FLAG antibody-conjugated resins and subsequently assayed by WB due to an overlap between CAMK-3 phospho-isoforms and nonspecific bands in the Phos tag gel.

Data in Fig. 2 and 3 are consistent with NCA-2 acting through kinases or phosphatases on FRQ, and the elevated calcium in the Δ*nca-2* mutant (36) might activate Ca^2+^-responsive kinases to overphosphorylate FRQ (Fig. 2F and G). CAMKs have been well documented to be activated by elevated intracellular Ca^2+^ and calmodulin. There are four *camk* genes (*camk-1* to *-4*) annotated in the *Neurospora* genome, and their catalytic domains are conserved despite a low overall identity of amino acid sequences (37). Expression of *camk-1* to *-4* genes moderately increases in the Δ*nca-2* mutant compared to their levels of expression in the WT across 28 h in the dark (Fig. S3). Among the four CAMKs, CAMK-1 has been reported to directly phosphorylate FRQ at multiple sites *in vitro*, although only a very subtle period defect was observed in the Δ*camk-1* mutant (18); however, in our hands, the Δ*camk-1* strain showed greatly reduced growth and was arrhythmic on race tubes (Fig. S4A), suggesting that prior data may have reflected a revertant strain. To further evaluate this and characterize roles for CAMKs, we made all combinations of *Δcamk* mutants, backcrossed these to the *C-box–luc* reporter, and assayed their clocks. We found that circadian periods of strains with individual or combinational knockouts of *camk* genes are indeed quite similar to that of the WT (Fig. S4B). To test whether the Δ*nca-2* mutant regulates the clock through *camk-1* to *-4*, the Δ*nca-2* mutant was backcrossed to mutants lacking *camk-1* to *-4*, and circadian periods were assayed by luciferase analyses. Interestingly, the Δ*camk-1*, *-3*, and *-4* mutants each showed the characteristic period shortening when in combination with the Δ*nca-2* mutant; however, the Δ*camk-2* Δ*nca-2* mutant showed the same circadian period as the *Δcamk-2* single mutant, with no additional shortening due to Δ*nca-2* (Fig. 4A), suggesting that *nca-2* and *camk-2* function in the same pathway to regulate the circadian period. Because in certain cases activated kinases not only phosphorylate their substrates but also actuate autophosphorylation in *cis* or in *trans*, phosphorylation on these kinases can be indicative of their activities. To test this, the phosphorylation status of CAMK-1 to -4 was determined by Western blotting using the 149:1 (acrylamide-bisacrylamide) Phos tag gel that has been used to resolve single phosphorylation events on WC-1 and WC-2 (10). CAMK-2 and -4 display similar phospho-profiles in the presence or absence of *nca-2*, while, interestingly, CAMK-1 and -3 in the Δ*nca-2* mutant undergo more phosphorylations than they do in the WT background (Fig. 4B), suggesting that their activities might be stimulated due to elevated calcium resulting from the absence of *nca-2*. Taken together, these data suggest that the elevated calcium concentration in the Δ*nca-2* mutant directly or indirectly activates CAMKs, which leads to hyper-phosphorylation of FRQ, thereby shortening the circadian period. The data further indicate that although intracellular calcium can influence periodicity through CAMKs, phosphorylation by CAMKs is not required for rhythmicity; it is modulatory.

10.1128/mBio.01493-21.3 FIG S3 Expression of *camk* genes in the WT and the Δ*nca-2* mutant. mRNA levels of *camk-1* to *-4* in the WT and the Δ*nca-2* mutant were assayed by RT-qPCR with samples grown under light or dark conditions as indicated. Levels of expression of *camk* genes were normalized to that of *rac-1.* FIG S3, PDF file, 0.2 MB Copyright © 2021 Wang et al. 2021 Wang et al. https://creativecommons.org/licenses/by/4.0/ This content is distributed under the terms of the Creative Commons Attribution 4.0 International license.

10.1128/mBio.01493-21.4 FIG S4 Individual or combinational deletion of *camk* genes does not dramatically impact the circadian period, as determined by the luciferase assay. (A) Mutants with individual *camk-1* to *-4* genes knocked out were assayed for circadian phenotypes by race tube analysis. Δ*camk-1* cultures in the top and middle tubes are true knockouts, while the one at the bottom is a typical revertant, as reported in reference 18. (B) Combinations of *Δcamk* mutations were tested by the luciferase assay. Download FIG S4, PDF file, 0.1 MB. Copyright © 2021 Wang et al. 2021 Wang et al. https://creativecommons.org/licenses/by/4.0/ This content is distributed under the terms of the Creative Commons Attribution 4.0 International license.

### Characterization of *nca-2*.

**FIG 5:**
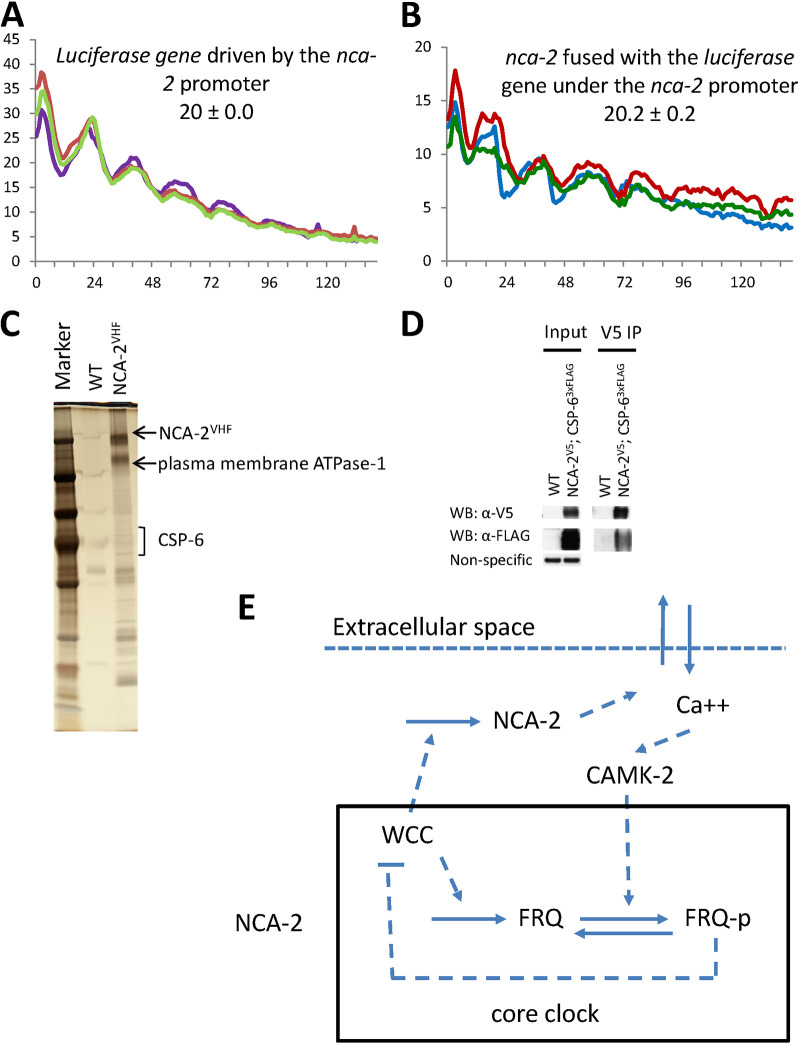
*nca-2* is a *ccg* and modulates both input to and output from the core clock. (A) The *nca-2* promoter fused to the *luciferase* gene was transformed to the *csr* locus, and luciferase signals were followed at 25°C in the dark. Periods (in hours) were determined as described in Materials and Methods and are reported ± SD (*n* = 3). (B) The *nca-2* open reading frame was fused to the 5′ end of the firefly *luciferase* gene, and the same assay as described for panel A was performed to trace the luciferase signal. (C) Representative silver-stained gel showing NCA-2^VHF^ and its interactome purified from a culture grown in the light. NCA-2^VHF^ and interactors were affinity purified, trichloroacetic acid (TCA) precipitated, and analyzed by mass spectrometry. (D) NCA-2 is tagged with a V5 tag, and one of its interactors, CSP-6, was tagged with a 3×FLAG tag. Coimmunoprecipitation was performed using V5 resin, and Western blotting was done with V5 and FLAG antibodies. (E) Working model for the roles of intracellular calcium and of *nca-2* in the circadian system. In the Δ*nca-2* mutant, increased calcium overactivates CAMKs, which induces FRQ overphosphorylation and thereby causes a faster-running clock; the circadian clock regulates the expression of the *nca-2* and *camk* genes.

In the *Neurospora* genome, transcription of ∼40% of coding genes is circadianly controlled directly or indirectly by the WCC-FFC oscillator (69, 70). We used transcriptional and translational fusions with the *luciferase* gene to see whether *nca-2* is a *ccg* (clock-controlled gene). First, the *nca-2* promoter was fused to the *luciferase* gene and transformed to the *csr* locus for real-time analysis of *nca-2* transcription, showing that transcription driven by the *nca-2* promoter is clearly rhythmic (Fig. 5A). Second, after fusing the *nca-2* coding sequence with the *luciferase* open reading frame (ORF), tracking the bioluminescent signal of NCA-2-LUC protein revealed that the NCA-2-LUC signal also oscillates in a typical circadian manner (Fig. 5B). These data indicate that calcium signaling in the cell might be regulated by the circadian clock through rhythmically transcribing and translating a calcium pump gene, *nca-2*. These data place NCA-2 in the larger cellular circadian system; levels of *nca-2* and NCA-2 expression are clock regulated, and NCA-2 activity, or a lack thereof, impacts circadian period length. To identify potential DNA elements conferring circadian transcription of *nca-2*, we searched rhythmic motifs derived from reference 69. These were identified as sequences that were overrepresented among rhythmically expressed genes. Interestingly, the first three of the four types of motifs identified in reference 69 are found in the *nca-2* promoter (1.7 kb upstream of ATG) (data not shown). However, we do not know what transcription factors (TFs) bind to these motifs; they do not appear in available databases, including the extensive catalogue of inferred sequence preferences of DNA-binding proteins (Cis-BP; http://cisbp.ccbr.utoronto.ca) (71) that covers >1,000 TFs from 131 species, including *Neurospora*. Although there were weak matches to the motifs, none of the matches were from *Neurospora* (data not shown).

Consistently with its role as a calcium exporter, NCA-2 is predicted to contain two calcium ATPase domains and a haloacid dehalogenase (HAD) domain (Fig. S5A). To understand the role of NCA-2 at a mechanistic level, we mapped the NCA-2 interactome by affinity purification. C-terminally V5-10×His-3×FLAG (VHF)-tagged NCA-2 was affinity purified under a nondenaturing condition (Fig. 5C), and its interacting proteins were identified by mass spectrometry. Among NCA-2’s interactors identified (Table S1) was the phosphatase CSP-6, whose interaction with NCA-2 was confirmed by immunoprecipitation (Fig. 5D). CSP-6 has been shown to control circadian output and WCC phosphorylations independently of the circadian feedback loop (72), suggesting that NCA-2 might have other roles relevant to CSP-6. Both the Δ*csp-6* mutant and the Δ*csp-6* Δ*nca-2* double mutant display an arrhythmic overt clock on race tubes (Fig. S5B), indicating that the Δ*nca-2* mutant is unable to rescue the output defect in the Δ*csp-6* mutant. Interestingly, however, while growing more slowly than the Δ*csp-6* mutant, the Δ*csp-6* Δ*nca-2* double mutant shows a period similar to that of the Δ*nca-2* mutant by the luciferase assay (Fig. S5C), suggesting that *nca-2* does not act through *csp-6* in controlling the pace of the core oscillator. All together, these data demonstrate that *nca-2* is a *ccg* and suggest that cellular calcium signaling might be regulated by the circadian clock via rhythmic expression of *nca-2*.

10.1128/mBio.01493-21.5 FIG S5 NCA-2 domain analyses and its relationship with *csp-6* on the clock. (A) Predicted domains in NCA-2 analyzed by the online tool SMART (http://smart.embl-heidelberg.de/smart/set_mode.cgi? NORMAL=1). (B and C) Race tube (B) and luciferase (C) analyses of the Δ*nca-2*, Δ*csp-6*, and Δ*csp-6* Δ*nca-2* mutants. Download FIG S5, PDF file, 0.2 MB. Copyright © 2021 Wang et al. 2021 Wang et al. https://creativecommons.org/licenses/by/4.0/ This content is distributed under the terms of the Creative Commons Attribution 4.0 International license.

10.1128/mBio.01493-21.7 TABLE S1 List of NCA-2 interactomes identified by tandem mass spectrometry from TCA-precipitated samples. Download Table S1, DOCX file, 0.04 MB. Copyright © 2021 Wang et al. 2021 Wang et al. https://creativecommons.org/licenses/by/4.0/ This content is distributed under the terms of the Creative Commons Attribution 4.0 International license.

### Downregulation of *calcineurin* does not influence the circadian period.

In a wide variety of eukaryotes, a prolonged increase in intracellular Ca^2+^ activates a calcium- and calmodulin-dependent serine/threonine protein phosphatase, calcineurin, which mediates the dephosphorylation of transcription factors, such as NFAT, to regulate gene expression (73–86). *calcineurin* (*ncu03833*) is an essential gene in Candida albicans and *Neurospora* (87, 88), so to determine whether *calcineurin* influences the circadian clock, we downregulated its expression by replacing its native promoter with the *qa-2* promoter, an inducible promoter activated by quinic acid (QA). In the absence of QA, WC-1 is undetectable and FRQ is barely seen in *qa-2-*driven *calcineurin* (Fig. S6A), consistent with a short period/arrhythmic clock observed in the strain (Fig. S6B). To better examine this, we assayed rhythmicity at extremely low levels of the inducer, i.e., levels just sufficient for rhythmicity (10^−8^ M QA), or at high levels at or above WT expression levels (10^−2^ M QA). We found that period length was not proportional to the level of *calcineurin* expression at levels supporting any rhythmicity and that even at vanishingly low *calcineurin* expression levels, the core oscillator displays a period similar to that of the WT, suggesting that the level of *calcineurin* does not determine the pace of the clock. This said, the severe reduction in WC-1 levels in the *qa-2*-driven *calcineurin* strain cultured without QA would be consistent with at least an indirect role for *calcineurin* in controlling WC-1 expression.

10.1128/mBio.01493-21.6 FIG S6 Downregulation of *calcineurin subunit B* has little influence on circadian period length across expression levels compatible with rhythmicity. The endogenous promoter of *calcineurin subunit B* was replaced by the *qa-2* promoter. (A) FRQ, WC-1, and WC-2 proteins were determined by Western blotting with specific antibodies. (B) *frq* transcription was tracked by *frq C-box–luc* at the *his-3* locus in the absence or presence of quinic acid (QA) at the concentration indicated. Download FIG S6, PDF file, 0.1 MB. Copyright © 2021 Wang et al. 2021 Wang et al. https://creativecommons.org/licenses/by/4.0/ This content is distributed under the terms of the Creative Commons Attribution 4.0 International license.

## DISCUSSION

In this study, we have identified *nca-2* as encoding a calcium pump involved in regulating circadian period length through CAMK-mediated FRQ phosphorylations. These data confirm that calcium signaling, a crucial regulatory pathway in mediating cellular and biochemical processes, must be well controlled for normal circadian period length determination. Most significantly, calcium signaling is now placed as an ancillary feedback loop within the larger circadian oscillatory system. The clock controls the expression of NCA-2—and thereby, intracellular calcium levels—and intracellular calcium, in turn, modulates the period length of the clock. In this regard, the larger *Neurospora* circadian system is regulated by calcium in a manner reminiscent of that seen in the mammalian brain (e.g., see reference 89). As prolonged activation of signaling pathways is wasteful and harmful to the cell, the elevated cytosolic calcium in the Δ*nca-2* mutant overactivates CAMKs, leading to FRQ hyper-phosphorylation and thereby causing a period defect (Fig. 5E). The involvement of intracellular Ca^2+^ in the circadian system is further nuanced by the finding that the expression of some *camk* genes is clock controlled (Fig. S3 and see references 69 and 70), so both the activator and effectors of calcium-induced regulation are clock-modulated and clock-affecting. This emphasizes the pervasive nature of both circadian and calcium control of the biology of the cell (Fig. 5E).

Among calcium-trafficking genes, *nca-2* encodes the major Ca^2+^ exporter (34). *Neurospora* encodes three transporter *nca* genes as well as the vacuolar calcium importer gene *cax*, but interestingly, only disruption of *nca-2* leads to a significant period change (Fig. 1A), suggesting that NCA-2 plays a major role in lowering cytosolic calcium. Consistently with this, the calcium level in the Δ*nca-2* mutant has been reported to rise ∼9.3 times, while it remains normal in the Δ*nca-1* or Δ*nca-3* mutant (36). It is possible that NCA-2 has higher affinity for Ca^2+^, is more abundant on the plasma membrane, or is more efficient in transporting calcium than the other two NCAs.

Temporal FRQ phosphorylation, the core pacemaking mechanism in the circadian feedback loop, is mediated by multiple kinases, including at least CKI, CKII, and CAMK-1 (9, 16, 18). Deletion of the *camk-2* gene prevents the high intracellular Ca^2+^ level from shortening the circadian period, indicating its dominant role in mediating the effect of calcium on the clock and making it a likely addition to the CAMKs active on the clock. Periods of several *frq* phosphorylation mutants, the *frq^S72A^*^,^ *^S73A^*^,^ *^S76A^*, *frq^S538A^*^,^ *^S540A^*, and *frq^S632A^*^,^ *^S634A^* mutants (Fig. 3), were not significantly altered in the background of the Δ*nca-2* mutant, and the domain where FRQ S72, S73, and S76 are located bears CAMK motifs (9), consistent with calcium-activated CAMK acting through these residues. Interestingly, although it is CAMK-1 that has been shown to directly phosphorylate FRQ *in vitro* (18), its loss here did not abrogate the effects of the loss of NCA-2. It may be that the phosphosites targeted by different CAMKs on FRQ are distinct and have different effects on rhythmicity. A freshly germinated Δ*camk-1* mutant displays a developmental defect (18, 37), whereas mutants with the other three *camk* genes knocked out individually grow as robustly as the WT (Fig. S4A). However, the growth defect of Δ*camk-1* strains appears to rapidly revert back to normal after a few rounds of inoculation of the Δ*camk-1* mutant on new slants (18), suggesting that other CAMKs might be able to gradually compensate for the loss of *camk-1* over time.

WCC can be phosphorylated at over 90 sites, and a small group of these is required for the closure of the circadian feedback loop (10). Interestingly, in the Δ*nca-2* mutant, WC-1 is hyper-phosphorylated and more abundant than in the WT despite a reduced *wc-1* RNA level (Fig. 1A); this finding is consistent with a “black widow” model in which site-specific phosphorylation of transcription activators makes them inactive in driving transcription but more stable (90). However, lacking key phosphoresidues determining the feedback loop closure, *wc-1* mutants, such as the *wc-1^S971A^*^,^ *^S988A^*^,^ *^S990A^*^,^ *^S992A^*^,^ *^S994A^*^,^ *^S995A^* and *wc-2^S394A^*^,^ *^S428A^*^,^ *^S429A^*^,^ *^S433A^*^,^ *^S435A^* mutants, show rhythms with short circadian period lengths due to an elevated activity of WCC (10), whereas *Δnca-2* strains bearing hyper-phosphorylated and more stable WC-1 also display a short period (Fig. 1 A and B and 2C and E). One possible explanation is that the hyper-phosphorylation of WC-1 in the Δ*nca-2* mutant occurs at residues regulating the circadian amplitude/output instead of at residues required for the feedback loop closure, while the period-shortening effect in the Δ*nca-2* mutant is caused by enhanced FRQ phosphorylation. WCC phosphoresidues can be briefly classified into two categories: the ones involved in the feedback loop closure and the other ones regulating the robustness of *frq* transcription (the amplitude reflecting the peak to trough in circadian cycles) (10). Key *wcc* phospho-mutants showed an additive effect with the Δ*nca-2* mutant on period length, suggesting that NCA-2 is not directly involved in the regulation of sites participating in feedback loop closure but instead regulates WCC phosphoresidues relevant to the circadian amplitude.

## MATERIALS AND METHODS

### Strains and culture conditions.

328-4 (*ras-1^bd^ A*) was used as a wild-type strain in the race tube analyses, and 661-4a (*ras-1^bd^* A), which bears the *frq C-box* fused to a codon-optimized *luciferase* gene at the *his-3* locus, served as the wild type in luciferase assays. *Neurospora* transformation was performed as previously reported (91, 92). Medium in the race tube analyses contained 1× Vogel’s salts, 0.17% arginine, 1.5% agar, 50 ng/ml biotin, and 0.1% glucose, and liquid culture medium (LCM) contained 1× Vogel’s salts, 0.5% arginine, 50 ng/ml biotin, and 2% glucose. Unless otherwise specified, race tubes were cultured in constant light for 16 to 24 h at 25°C to synchronize strains and then transferred to the dark at 25°C. The Vogeloid (10×) used to make the Ca^2+^-free medium in Fig. 1D contains 100 mM NH_4_Cl, 20 mM MgCl_2_·6H_2_O, 100 mM KCl, 20 mM methionine, 50 ng/ml biotin, and 0.1% glucose (36).

### Bioluminescence assays.

Luciferase assays were conducted as previously described (10). Briefly, strains with the *frq C-box*–*luciferase* transcriptional reporter at the *his-3* locus were grown in 96-well plates bearing 0.1% glucose race tube medium having luciferin in constant light overnight (16 to 24 h) at 25°C and then transferred to the dark at 25°C to start circadian cycles. Bioluminescent signals were tracked by a charge-coupled device (CCD) camera every hour for 5 or more days. Luciferase data were extracted using the NIH ImageJ software with a custom macro, and circadian period lengths were manually determined.

### Protein lysate and WB.

For Western blotting (WB), 15 mg of whole-cell protein lysate was loaded per lane on a 3 to 8% Tris-acetate or 6.5% Tris-glycine (bearing a Phos tag) SDS gel (92). Custom-raised antibodies against WC-1, WC-2, FRQ, and FRH have been described previously (93–95). V5 antibody (Thermo Pierce) and FLAG antibody (M2; Sigma-Aldrich) were diluted 1:5,000 for use as the primary antibody. To analyze the phosphorylation profiles of CAMKs, 20 μM Phos tag chemical (ApexBio) was added to the homemade 6.5% Tris-glycine SDS-PAGE gel bearing a ratio of 149:1 acrylamide to bisacrylamide (10).

### IP.

Immunoprecipitation (IP) was performed as previously described (91, 92). Briefly, 2 mg of total protein was incubated with 20 μl of V5 agarose (Sigma-Aldrich), with rotation at 4°C for 2 h. The agarose beads were washed with 1 ml of protein extraction buffer (50 mM HEPES [pH 7.4], 137 mM NaCl, 10% glycerol, 0.4% NP-40) twice and eluted with 50 μl of 5× SDS sample buffer at 99°C for 5 min.

### Other techniques.

RNA extraction, reverse transcription (RT), and quantitative PCR (qPCR) were conducted as previously reported (72, 91). V5-10×His-3×FLAG (VHF)-tagged NCA-2 was purified with the same method applied for isolation of C-terminal VHF-tagged WC-1, and mass spectrometry analyses were performed as previously described (72, 91). Data acquisition and analysis of luciferase runs were carried out as previously described (10).

10.1128/mBio.01493-21.8 TABLE S2 Primers sets used in the quantitative PCR. Download Table S2, DOCX file, 0.01 MB. Copyright © 2021 Wang et al. 2021 Wang et al. https://creativecommons.org/licenses/by/4.0/ This content is distributed under the terms of the Creative Commons Attribution 4.0 International license.
