# Supplementary figures and images for: Cellular Calcium Levels Influenced by NCA-2 Impact Circadian Period Determination in Neurospora
Source: mBio. 2021 Jun 29;12(3):e01493-21. doi: 10.1128/mBio.01493-21 (PMC8262947; doi:10.1128/mBio.01493-21)

# Figure S1

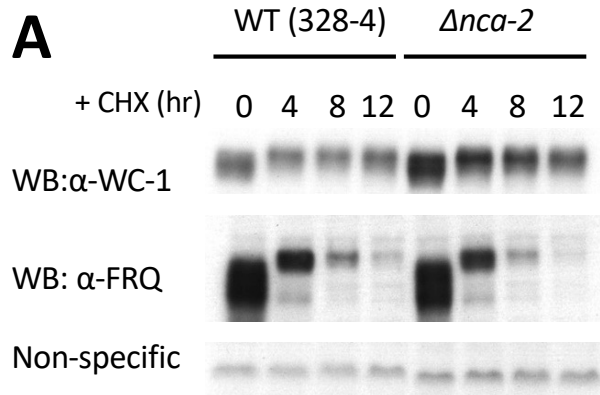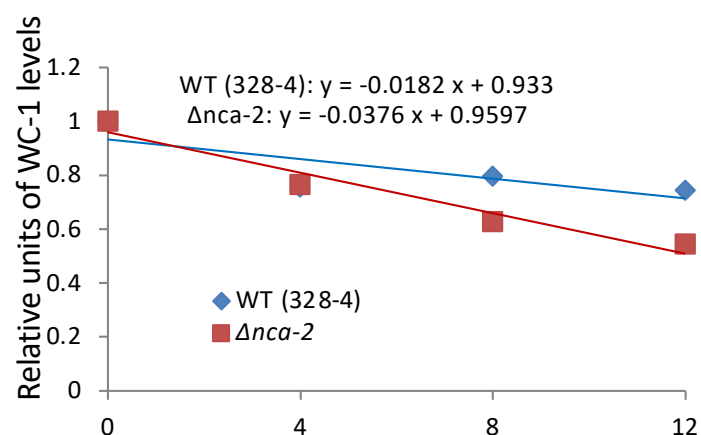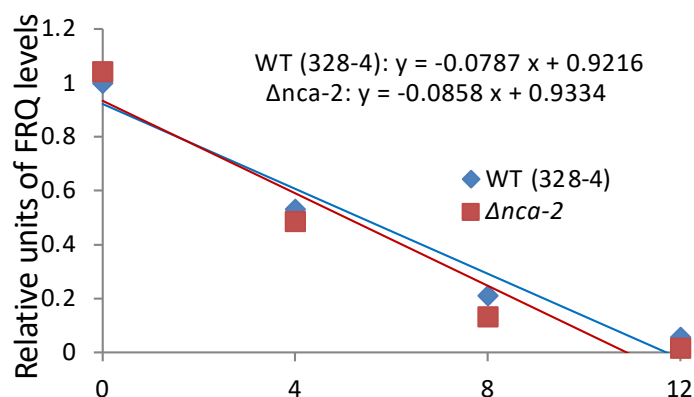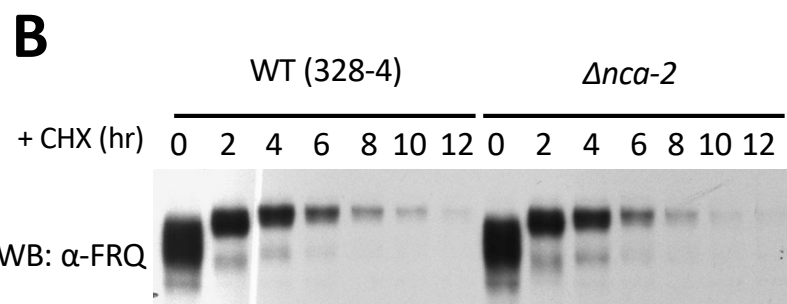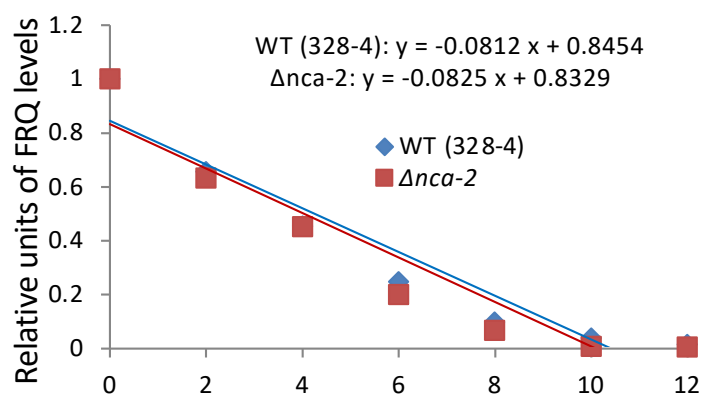

Supplement: FIG S1 [file mbio.01493-21-sf001.pdf]

# Figure S2

A

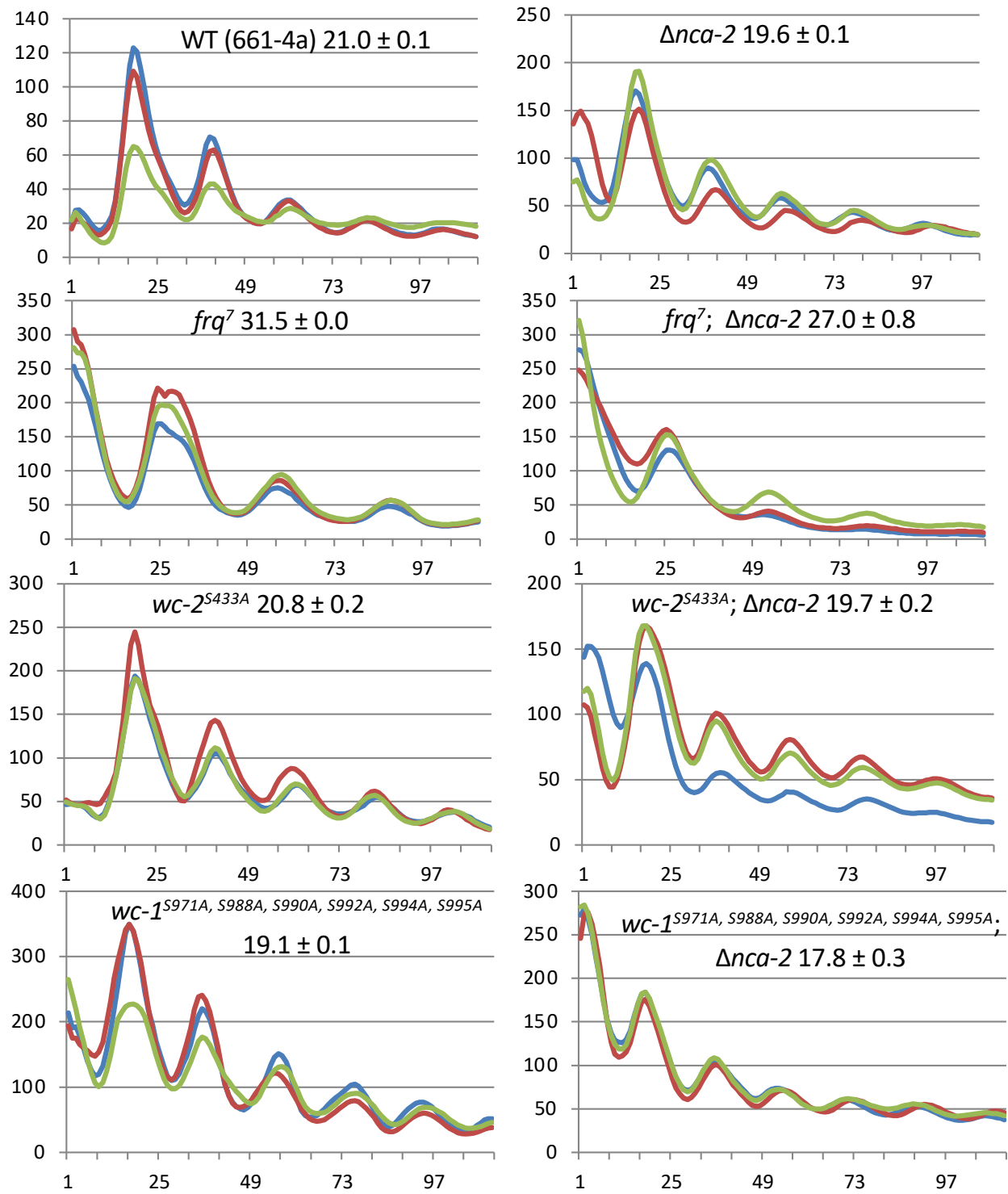

B

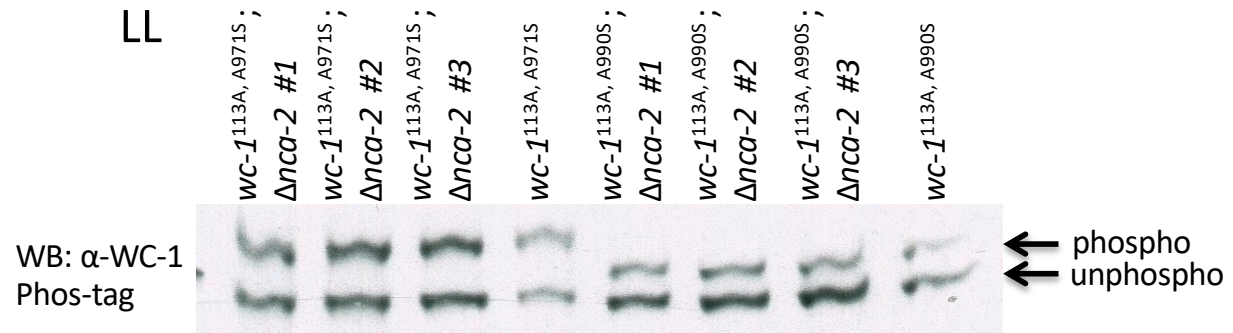

Supplement: FIG S2 [file mbio.01493-21-sf002.pdf]

# Figure S3

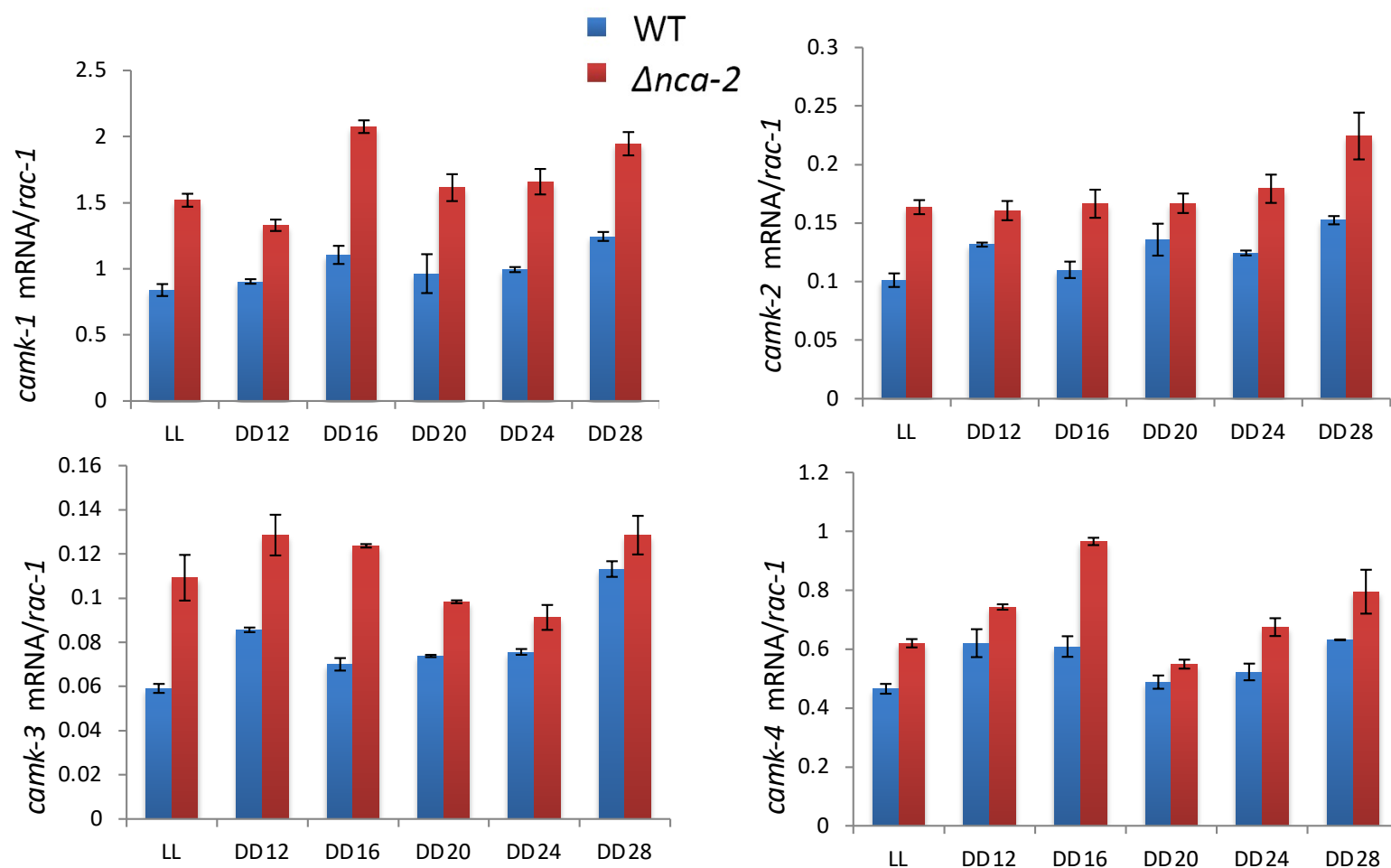

Supplement: FIG S3 [file mbio.01493-21-sf003.pdf]

# Figure S4

**A**

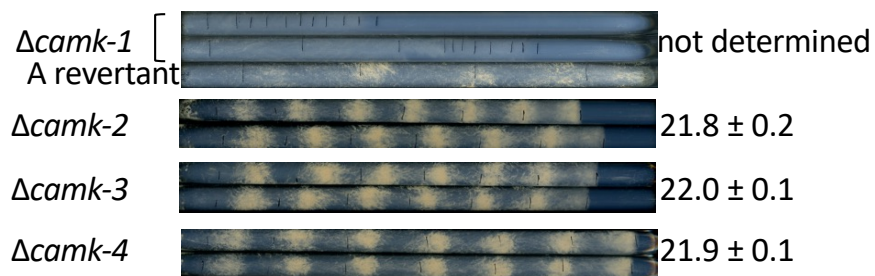

**B**

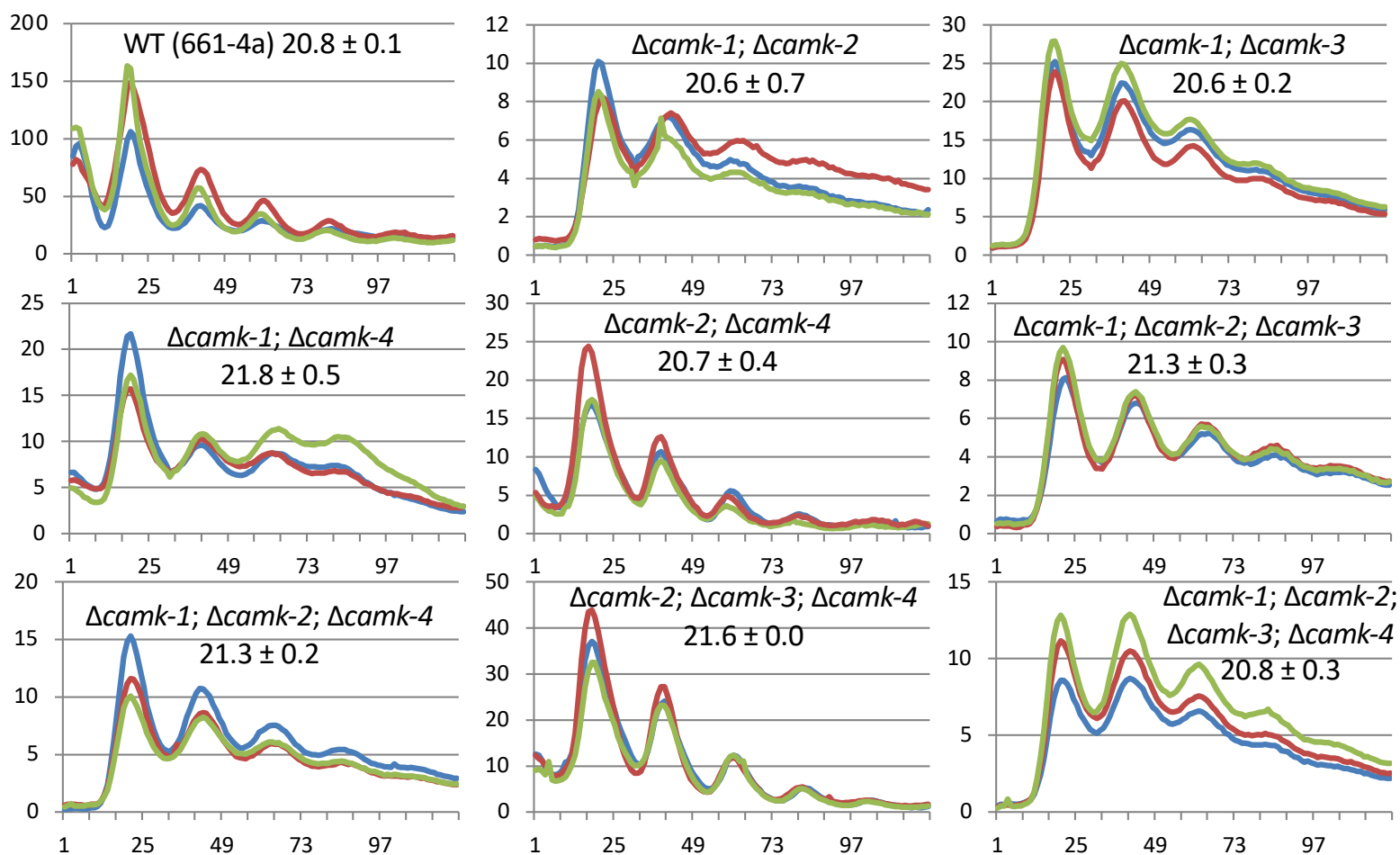

Supplement: FIG S4 [file mbio.01493-21-sf004.pdf]

# Figure S5

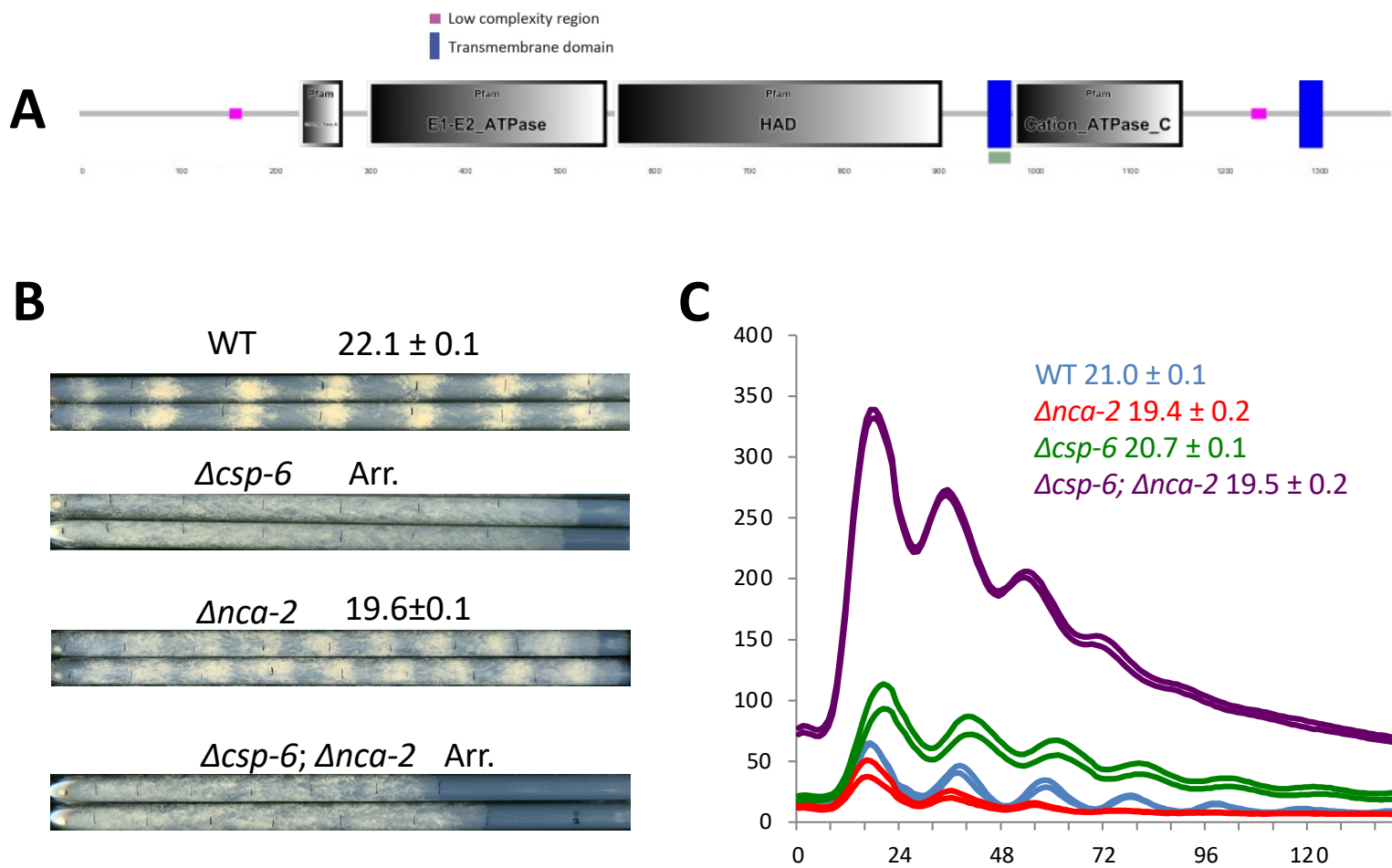

Supplement: FIG S5 [file mbio.01493-21-sf005.pdf]

# Figure S6

**A**

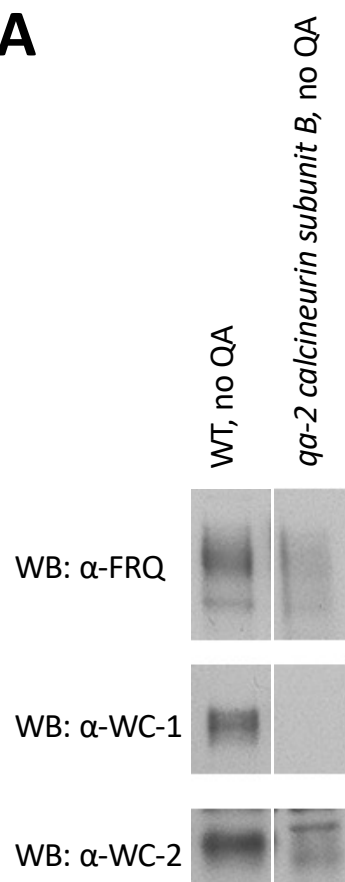

**B**

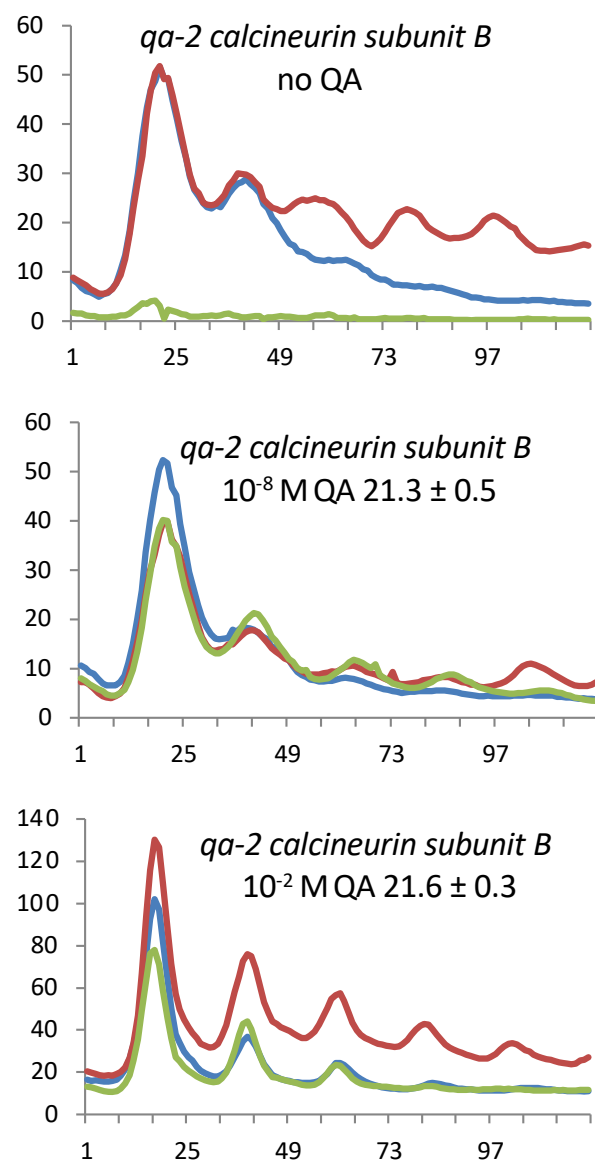

Supplement: FIG S6 [file mbio.01493-21-sf006.pdf]
