# Supplementary material for: Cellular Calcium Levels Influenced by NCA-2 Impact Circadian Period Determination in Neurospora
Source: mBio. 2021 Jun 29;12(3):e01493-21. doi: 10.1128/mBio.01493-21 (PMC8262947; doi:10.1128/mBio.01493-21)
Supplement: TABLE S1 [file mbio.01493-21-st001.docx]

**Table S1** Listed of NCA-2 interactome identified by tandem mass spectrometry from TCA-precipitated samples. Protein identification was determined by sequencing digested peptides and searching the *Neurospora* database: total peptide (tp): the number of distinct peptides belonging to the protein; unique peptide (up): peptides only existing in the protein (they may appear more than once in the same protein); unique match (um): unique peptides whose sequences only appear once in the same protein. The number of peptides derived from the same protein isolated from WT (untagged) and the tagged strain were compared to conclude the significance of their interactions with NCA-2. In no case were more than 5 tp, 4 up, or 4 um identified in the untagged control ms runs.

| name | accession | description | mol_weight | length | %_coverage | tp | up | um |
| --- | --- | --- | --- | --- | --- | --- | --- | --- |
| NCU04736T0 | NCU04736T0 | Neurospora crassa OR74A calcium P-type ATPase-2 (1450 aa) | 157757.4 | 1449 | 49.96549344 | 304 | 104 | 101 |
| NCU01680T0 | NCU01680T0 | Neurospora crassa OR74A plasma membrane ATPase-1 (921 aa) | 99805.82 | 920 | 22.5 | 40 | 30 | 28 |
| NCU00585T0 | NCU00585T0 | Neurospora crassa OR74A albino-2 (603 aa) | 68865.31 | 602 | 34.55149502 | 36 | 30 | 30 |
| NCU04173T0 | NCU04173T0 | Neurospora crassa OR74A actin (376 aa) | 41562.9 | 375 | 40 | 24 | 18 | 0 |
| NCU04173T1 | NCU04173T1 | Neurospora crassa OR74A actin (376 aa) | 41562.9 | 375 | 40 | 24 | 18 | 0 |
| NCU00489T0 | NCU00489T0 | Neurospora crassa OR74A cytoplasmic ribosomal protein-10 (263 aa) | 28630.92 | 262 | 70.99236641 | 23 | 19 | 19 |
| NCU01767T0 | NCU01767T0 | Neurospora crassa OR74A phosphatase 2C family protein (623 aa) | 67123.2 | 622 | 35.20900322 | 23 | 19 | 19 |
| NCU07414T0 | NCU07414T0 | Neurospora crassa OR74A hypothetical protein similar to protein mitochondrial targeting protein Mas5 (415 aa) | 45270.35 | 414 | 29.71014493 | 21 | 16 | 16 |
| NCU05390T0 | NCU05390T0 | Neurospora crassa OR74A mitochondrial phosphate carrier protein (320 aa) | 33281.34 | 319 | 44.20062696 | 18 | 16 | 15 |
| NCU03297T0 | NCU03297T0 | Neurospora crassa OR74A cytochrome c peroxidase (359 aa) | 39774.86 | 358 | 37.98882682 | 17 | 13 | 0 |
| NCU03297T1 | NCU03297T1 | Neurospora crassa OR74A cytochrome c peroxidase (359 aa) | 39774.86 | 358 | 37.98882682 | 17 | 13 | 0 |
| NCU03297T2 | NCU03297T2 | Neurospora crassa OR74A cytochrome c peroxidase (359 aa) | 39774.86 | 358 | 37.98882682 | 17 | 13 | 0 |
| NCU01528T0 | NCU01528T0 | Neurospora crassa OR74A glyceraldehyde-3-phosphate dehydrogenase-1 (339 aa) | 36251.52 | 338 | 31.65680473 | 15 | 12 | 12 |
| NCU02003T0 | NCU02003T0 | Neurospora crassa OR74A translation elongation factor-1 (461 aa) | 49623.06 | 460 | 28.69565217 | 15 | 12 | 12 |
| NCU03305T0 | NCU03305T0 | Neurospora crassa OR74A calcium P-type ATPase-1 (998 aa) | 107589.4 | 997 | 15.24573721 | 15 | 14 | 14 |
| NCU06265T0 | NCU06265T0 | Neurospora crassa OR74A hypothetical protein (888 aa) | 98345.78 | 887 | 18.71476888 | 15 | 15 | 15 |
| NCU09602T0 | NCU09602T0 | Neurospora crassa OR74A heat shock protein 70-1 (647 aa) | 70492 | 646 | 23.68421053 | 15 | 15 | 13 |
| NCU01754T0 | NCU01754T0 | Neurospora crassa OR74A alcohol dehydrogenase-1 (354 aa) | 37401.25 | 353 | 39.94334278 | 14 | 14 | 7 |
| NCU00465T0 | NCU00465T0 | Neurospora crassa OR74A chaperone dnaJ (424 aa) | 46489.79 | 423 | 23.64066194 | 13 | 12 | 0 |
| NCU00465T1 | NCU00465T1 | Neurospora crassa OR74A chaperone dnaJ (424 aa) | 46489.79 | 423 | 23.64066194 | 13 | 12 | 0 |
| NCU06220T0 | NCU06220T0 | Neurospora crassa OR74A hypothetical protein (501 aa) | 57860.6 | 500 | 21.8 | 12 | 12 | 0 |
| NCU06220T1 | NCU06220T1 | Neurospora crassa OR74A hypothetical protein (462 aa) | 53531.71 | 461 | 23.64425163 | 12 | 12 | 0 |
| NCU02075T0 | NCU02075T0 | Neurospora crassa OR74A heat shock protein 70-2 (586 aa) | 63452.02 | 585 | 21.70940171 | 11 | 11 | 11 |
| NCU03982T0 | NCU03982T0 | Neurospora crassa OR74A glucose regulated protein 78 (662 aa) | 72269.03 | 661 | 17.85173979 | 11 | 11 | 10 |
| NCU07465T0 | NCU07465T0 | Neurospora crassa OR74A mitochondrial phosphate carrier protein 2 (383 aa) | 41786.09 | 382 | 23.56020942 | 11 | 10 | 0 |
| NCU07465T1 | NCU07465T1 | Neurospora crassa OR74A mitochondrial phosphate carrier protein 2 (383 aa) | 41786.09 | 382 | 23.56020942 | 11 | 10 | 0 |
| NCU09132T0 | NCU09132T0 | Neurospora crassa OR74A tubulin alpha-1 (455 aa) | 50206.81 | 454 | 24.66960352 | 11 | 11 | 9 |
| NCU09475T0 | NCU09475T0 | Neurospora crassa OR74A 40s ribosomal protein s5 (214 aa) | 23647.39 | 213 | 41.31455399 | 11 | 10 | 10 |
| NCU00618T0 | NCU00618T0 | Neurospora crassa OR74A 40S ribosomal protein S27 (83 aa) | 8873.569 | 82 | 50 | 10 | 7 | 7 |
| NCU06743T0 | NCU06743T0 | Neurospora crassa OR74A hypothetical protein (164 aa) | 18402.29 | 163 | 33.74233129 | 10 | 8 | 8 |
| NCU04054T0 | NCU04054T0 | Neurospora crassa OR74A Benomyl resistant (448 aa) | 49817.97 | 447 | 14.09395973 | 9 | 8 | 8 |
| NCU04553T0 | NCU04553T0 | Neurospora crassa OR74A ubiquitin/cytoplasmic ribosomal protein-6 (155 aa) | 17709.46 | 154 | 34.41558442 | 9 | 6 | 0 |
| NCU04553T1 | NCU04553T1 | Neurospora crassa OR74A ubiquitin/cytoplasmic ribosomal protein-6 (155 aa) | 17709.46 | 154 | 34.41558442 | 9 | 6 | 0 |
| NCU05275T0 | NCU05275T0 | Neurospora crassa OR74A ubiquitin fusion protein (129 aa) | 14609.9 | 128 | 41.40625 | 9 | 6 | 0 |
| NCU05995T0 | NCU05995T0 | Neurospora crassa OR74A ubiquitin (306 aa) | 34426.43 | 305 | 17.37704918 | 9 | 6 | 0 |
| NCU06210T0 | NCU06210T0 | Neurospora crassa OR74A hypothetical protein (151 aa) | 15951.76 | 150 | 46 | 9 | 9 | 9 |
| NCU08045T0 | NCU08045T0 | Neurospora crassa OR74A choline-1 (966 aa) | 109464.7 | 965 | 9.948186528 | 9 | 9 | 9 |
| NCU09477T0 | NCU09477T0 | Neurospora crassa OR74A ADP, ATP carrier protein (314 aa) | 33848.67 | 313 | 26.19808307 | 9 | 9 | 2 |
| NCU02514T0 | NCU02514T0 | Neurospora crassa OR74A ATPase-1 (552 aa) | 59467.23 | 551 | 15.78947368 | 8 | 8 | 8 |
| NCU03112T0 | NCU03112T0 | Neurospora crassa OR74A NADH-cytochrome b5 reductase 2 (344 aa) | 37599.69 | 343 | 17.49271137 | 8 | 7 | 7 |
| NCU07232T0 | NCU07232T0 | Neurospora crassa OR74A hsp30-like protein (280 aa) | 30943.9 | 279 | 19.35483871 | 8 | 6 | 6 |
| NCU00829T0 | NCU00829T0 | Neurospora crassa OR74A ferric reductase (680 aa) | 75329.37 | 679 | 11.634757 | 7 | 7 | 7 |
| NCU01754T1 | NCU01754T1 | Neurospora crassa OR74A alcohol dehydrogenase-1 (328 aa) | 34766.42 | 327 | 23.85321101 | 7 | 7 | 0 |
| NCU06047T0 | NCU06047T0 | Neurospora crassa OR74A 40S ribosomal protein S2 (266 aa) | 28720.59 | 265 | 20.75471698 | 7 | 7 | 7 |
| NCU08340T0 | NCU08340T0 | Neurospora crassa OR74A ADP-ribosylation factor 1 (186 aa) | 20947.89 | 185 | 38.37837838 | 7 | 7 | 0 |
| NCU08340T1 | NCU08340T1 | Neurospora crassa OR74A ADP-ribosylation factor 1 (186 aa) | 20947.89 | 185 | 38.37837838 | 7 | 7 | 0 |
| NCU09468T0 | NCU09468T0 | Neurospora crassa OR74A tubulin alpha-2 (450 aa) | 49916.14 | 449 | 17.14922049 | 7 | 7 | 5 |
| NCU09477T1 | NCU09477T1 | Neurospora crassa OR74A ADP, ATP carrier protein (221 aa) | 24071.52 | 220 | 28.18181818 | 7 | 7 | 0 |
| NCU11181T0 | NCU11181T0 | Neurospora crassa OR74A Ras superfamily GTPase (190 aa) | 21551.28 | 189 | 44.44444444 | 7 | 7 | 7 |
| NCU03757T0 | NCU03757T0 | Neurospora crassa OR74A 60S ribosomal protein L4-A (362 aa) | 38772.08 | 361 | 17.45152355 | 6 | 6 | 6 |
| NCU05154T0 | NCU05154T0 | Neurospora crassa OR74A calcium P-type ATPase-3 (1153 aa) | 126230.6 | 1152 | 1.822916667 | 6 | 3 | 0 |
| NCU06986T0 | NCU06986T0 | Neurospora crassa OR74A DUF221 domain-containing protein (903 aa) | 101179.2 | 902 | 8.425720621 | 6 | 6 | 0 |
| NCU06986T1 | NCU06986T1 | Neurospora crassa OR74A DUF221 domain-containing protein (903 aa) | 101179.2 | 902 | 8.425720621 | 6 | 6 | 0 |
| NCU08620T0 | NCU08620T0 | Neurospora crassa OR74A 40S ribosomal protein S16 (143 aa) | 15695.69 | 142 | 35.21126761 | 6 | 6 | 6 |
| NCU00475T0 | NCU00475T0 | Neurospora crassa OR74A 40S ribosomal protein S18 (157 aa) | 17739.67 | 156 | 30.12820513 | 5 | 5 | 5 |
| NCU00673T0 | NCU00673T0 | Neurospora crassa OR74A serine protease p2 (533 aa) | 56713.17 | 532 | 8.646616541 | 5 | 5 | 5 |
| NCU01487T0 | NCU01487T0 | Neurospora crassa OR74A hypothetical protein (476 aa) | 52658.62 | 475 | 13.05263158 | 5 | 5 | 5 |
| NCU01633T0 | NCU01633T0 | Neurospora crassa OR74A hexose transporter HXT13 (533 aa) | 57936.72 | 532 | 10.15037594 | 5 | 4 | 4 |
| NCU03498T0 | NCU03498T0 | Neurospora crassa OR74A Lcc3 (694 aa) | 75045.98 | 693 | 8.080808081 | 5 | 5 | 5 |
| NCU03611T0 | NCU03611T0 | Neurospora crassa OR74A chitin synthase-1 (918 aa) | 102608.9 | 917 | 6.324972737 | 5 | 5 | 5 |
| NCU06431T0 | NCU06431T0 | Neurospora crassa OR74A 40S ribosomal protein S22 (131 aa) | 14792.95 | 130 | 33.84615385 | 5 | 4 | 0 |
| NCU06431T1 | NCU06431T1 | Neurospora crassa OR74A 40S ribosomal protein S22 (131 aa) | 14792.95 | 130 | 33.84615385 | 5 | 4 | 0 |
| NCU07830T0 | NCU07830T0 | Neurospora crassa OR74A cytoplasmic ribosomal protein-2 (151 aa) | 15969.44 | 150 | 40 | 5 | 5 | 5 |
| NCU09345T0 | NCU09345T0 | Neurospora crassa OR74A no message in thiamine-1 (343 aa) | 38156.29 | 342 | 11.40350877 | 5 | 5 | 0 |
| NCU09345T1 | NCU09345T1 | Neurospora crassa OR74A no message in thiamine-1 (343 aa) | 38156.29 | 342 | 11.40350877 | 5 | 5 | 0 |
| NCU09345T2 | NCU09345T2 | Neurospora crassa OR74A no message in thiamine-1 (343 aa) | 38156.29 | 342 | 11.40350877 | 5 | 5 | 0 |
| NCU02267T0 | NCU02267T0 | Neurospora crassa OR74A mitochondrial protein Fmp25 (607 aa) | 66206.54 | 606 | 6.435643564 | 4 | 4 | 0 |
| NCU02267T1 | NCU02267T1 | Neurospora crassa OR74A mitochondrial protein Fmp25 (607 aa) | 66206.54 | 606 | 6.435643564 | 4 | 4 | 0 |
| NCU02811T0 | NCU02811T0 | Neurospora crassa OR74A hyphal anastamosis-8 (527 aa) | 57655.21 | 526 | 8.745247148 | 4 | 4 | 4 |
| NCU05046T0 | NCU05046T0 | Neurospora crassa OR74A E1-E2 ATPase-1 (1122 aa) | 121561.4 | 1121 | 3.746654773 | 4 | 4 | 4 |
| NCU05259T0 | NCU05259T0 | Neurospora crassa OR74A acyl-CoA desaturase 1 (478 aa) | 53683.67 | 477 | 8.595387841 | 4 | 4 | 4 |
| NCU05453T0 | NCU05453T0 | Neurospora crassa OR74A ER to Golgi transporter (596 aa) | 64739.99 | 595 | 6.218487395 | 4 | 4 | 4 |
| NCU06132T0 | NCU06132T0 | Neurospora crassa OR74A siderophore iron transporter (598 aa) | 65921.18 | 597 | 7.370184255 | 4 | 4 | 4 |
| NCU06783T0 | NCU06783T0 | Neurospora crassa OR74A ATP citrate lyase (488 aa) | 52918.56 | 487 | 9.034907598 | 4 | 4 | 0 |
| NCU06783T1 | NCU06783T1 | Neurospora crassa OR74A ATP citrate lyase (488 aa) | 52918.56 | 487 | 9.034907598 | 4 | 4 | 0 |
| NCU07807T0 | NCU07807T0 | Neurospora crassa OR74A fructose bisphosphate aldolase (363 aa) | 39846.23 | 362 | 14.08839779 | 4 | 4 | 0 |
| NCU07807T1 | NCU07807T1 | Neurospora crassa OR74A fructose bisphosphate aldolase (363 aa) | 39846.23 | 362 | 14.08839779 | 4 | 4 | 0 |
| NCU07914T0 | NCU07914T0 | Neurospora crassa OR74A phosphoglycerate kinase (419 aa) | 45010.74 | 418 | 15.07177033 | 4 | 4 | 4 |
| NCU07965T0 | NCU07965T0 | Neurospora crassa OR74A dolichol-phosphate mannosyltransferase (245 aa) | 27067.03 | 244 | 16.80327869 | 4 | 4 | 4 |
| NCU09269T0 | NCU09269T0 | Neurospora crassa OR74A ran-like (216 aa) | 24242.28 | 215 | 16.27906977 | 4 | 4 | 0 |
| NCU09269T1 | NCU09269T1 | Neurospora crassa OR74A ran-like (216 aa) | 24242.28 | 215 | 16.27906977 | 4 | 4 | 0 |
| NCU09527T0 | NCU09527T0 | Neurospora crassa OR74A hypothetical protein (373 aa) | 40686.58 | 372 | 12.3655914 | 4 | 4 | 4 |
| NCU00573T0 | NCU00573T0 | Neurospora crassa OR74A hypothetical protein (749 aa) | 81597.48 | 748 | 3.609625668 | 3 | 3 | 3 |
| NCU01317T0 | NCU01317T0 | Neurospora crassa OR74A 60S ribosomal protein L12 (166 aa) | 17664.62 | 165 | 23.03030303 | 3 | 3 | 3 |
| NCU01735T0 | NCU01735T0 | Neurospora crassa OR74A hypothetical protein (293 aa) | 32796.22 | 292 | 13.69863014 | 3 | 3 | 0 |
| NCU01735T1 | NCU01735T1 | Neurospora crassa OR74A hypothetical protein (293 aa) | 32796.22 | 292 | 13.69863014 | 3 | 3 | 0 |
| NCU01957T0 | NCU01957T0 | Neurospora crassa OR74A AR2 (323 aa) | 35731.57 | 322 | 5.900621118 | 3 | 2 | 0 |
| NCU01977T0 | NCU01977T0 | Neurospora crassa OR74A urea transporter (537 aa) | 57471.9 | 536 | 5.597014925 | 3 | 3 | 1 |
| NCU02181T0 | NCU02181T0 | Neurospora crassa OR74A 40S ribosomal protein S4 (262 aa) | 29559.92 | 261 | 12.64367816 | 3 | 3 | 3 |
| NCU02193T0 | NCU02193T0 | Neurospora crassa OR74A cellular filament polypeptide (571 aa) | 62205.13 | 570 | 4.912280702 | 3 | 3 | 0 |
| NCU02193T1 | NCU02193T1 | Neurospora crassa OR74A cellular filament polypeptide (549 aa) | 59757.92 | 548 | 5.109489051 | 3 | 3 | 0 |
| NCU03102T0 | NCU03102T0 | Neurospora crassa OR74A 40S ribosomal protein S11 (162 aa) | 18399.07 | 161 | 17.39130435 | 3 | 3 | 3 |
| NCU03125T0 | NCU03125T0 | Neurospora crassa OR74A NIMA-interacting protein TinC (796 aa) | 89506.77 | 795 | 3.522012579 | 3 | 3 | 3 |
| NCU03393T0 | NCU03393T0 | Neurospora crassa OR74A ribosome-associated protein-1 (291 aa) | 31462.76 | 290 | 11.37931034 | 3 | 3 | 3 |
| NCU06110T0 | NCU06110T0 | Neurospora crassa OR74A thiazole biosynthetic enzyme (345 aa) | 36822.62 | 344 | 5.523255814 | 3 | 2 | 0 |
| NCU06110T1 | NCU06110T1 | Neurospora crassa OR74A thiazole biosynthetic enzyme (345 aa) | 36822.62 | 344 | 5.523255814 | 3 | 2 | 0 |
| NCU06110T2 | NCU06110T2 | Neurospora crassa OR74A thiazole biosynthetic enzyme (345 aa) | 36822.62 | 344 | 5.523255814 | 3 | 2 | 0 |
| NCU06110T3 | NCU06110T3 | Neurospora crassa OR74A thiazole biosynthetic enzyme (345 aa) | 36822.62 | 344 | 5.523255814 | 3 | 2 | 0 |
| NCU06207T0 | NCU06207T0 | Neurospora crassa OR74A C-5 sterol desaturase (345 aa) | 40618.2 | 344 | 11.62790698 | 3 | 3 | 3 |
| NCU06606T0 | NCU06606T0 | Neurospora crassa OR74A iron-sulfur subunit-1 (232 aa) | 24736.64 | 231 | 12.98701299 | 3 | 3 | 3 |
| NCU08500T0 | NCU08500T0 | Neurospora crassa OR74A 40S ribosomal protein S8 (203 aa) | 22979.13 | 202 | 15.84158416 | 3 | 3 | 0 |
| NCU08500T1 | NCU08500T1 | Neurospora crassa OR74A 40S ribosomal protein S8 (203 aa) | 22979.13 | 202 | 15.84158416 | 3 | 3 | 0 |
| NCU08949T0 | NCU08949T0 | Neurospora crassa OR74A hypothetical protein (170 aa) | 18113.35 | 169 | 16.56804734 | 3 | 2 | 1 |
| NCU08989T0 | NCU08989T0 | Neurospora crassa OR74A ADP-ribosylation factor 1 (184 aa) | 20345.32 | 183 | 14.75409836 | 3 | 3 | 3 |
| NCU00072T0 | NCU00072T0 | Neurospora crassa OR74A hypothetical protein (373 aa) | 39590.1 | 372 | 7.52688172 | 2 | 2 | 2 |
| NCU00830T0 | NCU00830T0 | Neurospora crassa OR74A ctr copper transporter (255 aa) | 28293 | 254 | 8.267716535 | 2 | 2 | 2 |
| NCU01484T0 | NCU01484T0 | Neurospora crassa OR74A rho-type GTPase-1 (196 aa) | 21939.35 | 195 | 11.79487179 | 2 | 2 | 2 |
| NCU01552T0 | NCU01552T0 | Neurospora crassa OR74A ribosomal protein S28 (146 aa) | 15881.75 | 145 | 12.4137931 | 2 | 2 | 0 |
| NCU01552T1 | NCU01552T1 | Neurospora crassa OR74A ribosomal protein S28 (146 aa) | 15881.75 | 145 | 12.4137931 | 2 | 2 | 0 |
| NCU01827T0 | NCU01827T0 | Neurospora crassa OR74A 60S ribosomal protein L27 (136 aa) | 15694.55 | 135 | 15.55555556 | 2 | 2 | 2 |
| NCU01977T1 | NCU01977T1 | Neurospora crassa OR74A urea transporter (399 aa) | 42612.94 | 398 | 5.527638191 | 2 | 2 | 0 |
| NCU02280T0 | NCU02280T0 | Neurospora crassa OR74A NADH:ubiquinone oxidoreductase 21.3b (201 aa) | 21317.87 | 200 | 11 | 2 | 2 | 2 |
| NCU02509T0 | NCU02509T0 | Neurospora crassa OR74A 60S ribosomal protein L11 (175 aa) | 20053.58 | 174 | 12.06896552 | 2 | 2 | 2 |
| NCU02618T0 | NCU02618T0 | Neurospora crassa OR74A peroxin 13 (458 aa) | 47312.29 | 457 | 5.470459519 | 2 | 2 | 2 |
| NCU02660T0 | NCU02660T0 | Neurospora crassa OR74A DUF962 domain-containing protein (191 aa) | 21140.32 | 190 | 8.947368421 | 2 | 2 | 2 |
| NCU03150T0 | NCU03150T0 | Neurospora crassa OR74A 60S ribosomal protein L24 (157 aa) | 17582.81 | 156 | 10.25641026 | 2 | 2 | 2 |
| NCU03362T0 | NCU03362T0 | Neurospora crassa OR74A steroid alpha reductase (317 aa) | 35670.86 | 316 | 5.063291139 | 2 | 2 | 2 |
| NCU03561T0 | NCU03561T0 | Neurospora crassa OR74A mitochondrial carrier protein (305 aa) | 33022.55 | 304 | 6.907894737 | 2 | 2 | 2 |
| NCU03989T0 | NCU03989T0 | Neurospora crassa OR74A ADP,ATP carrier protein (339 aa) | 36939.15 | 338 | 5.029585799 | 2 | 2 | 2 |
| NCU05225T0 | NCU05225T0 | Neurospora crassa OR74A NADH dehydrogenase 64 (674 aa) | 75783.35 | 673 | 3.714710253 | 2 | 2 | 2 |
| NCU05430T0 | NCU05430T0 | Neurospora crassa OR74A ATPase-2 (520 aa) | 55480.93 | 519 | 4.046242775 | 2 | 2 | 2 |
| NCU05514T0 | NCU05514T0 | Neurospora crassa OR74A golgi membrane protein (339 aa) | 34423.89 | 338 | 5.029585799 | 2 | 2 | 2 |
| NCU06226T0 | NCU06226T0 | Neurospora crassa OR74A 60S ribosomal protein L25 (157 aa) | 17090.62 | 156 | 17.30769231 | 2 | 2 | 2 |
| NCU06402T0 | NCU06402T0 | Neurospora crassa OR74A C-4 methylsterol oxidase (307 aa) | 35564.93 | 306 | 6.209150327 | 2 | 2 | 0 |
| NCU06402T1 | NCU06402T1 | Neurospora crassa OR74A C-4 methylsterol oxidase (261 aa) | 30368.35 | 260 | 7.307692308 | 2 | 2 | 0 |
| NCU06518T0 | NCU06518T0 | Neurospora crassa OR74A NADH-cytochrome b5 reductase 2 (327 aa) | 35397.62 | 326 | 6.748466258 | 2 | 2 | 2 |
| NCU06892T0 | NCU06892T0 | Neurospora crassa OR74A 40S ribosomal protein S20 (118 aa) | 13199.28 | 117 | 16.23931624 | 2 | 2 | 2 |
| NCU07014T0 | NCU07014T0 | Neurospora crassa OR74A cytoplasmic ribosomal protein-3 (147 aa) | 16914.06 | 146 | 13.69863014 | 2 | 2 | 2 |
| NCU07173T0 | NCU07173T0 | Neurospora crassa OR74A ADP-ribosylation factor 6 (185 aa) | 20815.94 | 184 | 9.782608696 | 2 | 2 | 1 |
| NCU07366T0 | NCU07366T0 | Neurospora crassa OR74A glucosamine-fructose-6-phosphate aminotransferase (701 aa) | 77896.04 | 700 | 2.142857143 | 2 | 2 | 2 |
| NCU07700T0 | NCU07700T0 | Neurospora crassa OR74A colonial temperature-sensitive-3 (845 aa) | 93185.28 | 844 | 1.895734597 | 2 | 2 | 2 |
| NCU08949T1 | NCU08949T1 | Neurospora crassa OR74A hypothetical protein (151 aa) | 16134.4 | 150 | 6.666666667 | 2 | 1 | 0 |
| NCU08949T2 | NCU08949T2 | Neurospora crassa OR74A hypothetical protein (151 aa) | 16134.4 | 150 | 6.666666667 | 2 | 1 | 0 |
| NCU09109T0 | NCU09109T0 | Neurospora crassa OR74A 60S ribosomal protein L33 (110 aa) | 12152.57 | 109 | 12.8440367 | 2 | 2 | 2 |
| NCU10042T0 | NCU10042T0 | Neurospora crassa OR74A embden-meyerhof pathway-7 (439 aa) | 47546.71 | 438 | 5.251141553 | 2 | 2 | 0 |
| NCU10042T1 | NCU10042T1 | Neurospora crassa OR74A embden-meyerhof pathway-7 (439 aa) | 47546.71 | 438 | 5.251141553 | 2 | 2 | 0 |
| NCU00018T0 | NCU00018T0 | Neurospora crassa OR74A cell division control protein Cdc48 (825 aa) | 90497.08 | 824 | 1.45631068 | 1 | 1 | 0 |
| NCU00018T1 | NCU00018T1 | Neurospora crassa OR74A cell division control protein Cdc48 (825 aa) | 90497.08 | 824 | 1.45631068 | 1 | 1 | 0 |
| NCU00030T0 | NCU00030T0 | Neurospora crassa OR74A mitochondrial nuclease (333 aa) | 36482.49 | 332 | 3.012048193 | 1 | 1 | 1 |
| NCU00182T0 | NCU00182T0 | Neurospora crassa OR74A integral membrane protein (555 aa) | 62179.37 | 554 | 2.346570397 | 1 | 1 | 1 |
| NCU00265T0 | NCU00265T0 | Neurospora crassa OR74A hypothetical protein (315 aa) | 31272.3 | 314 | 2.229299363 | 1 | 1 | 1 |
| NCU00413T0 | NCU00413T0 | Neurospora crassa OR74A 60S ribosomal protein L2 (255 aa) | 27321.71 | 254 | 3.149606299 | 1 | 1 | 1 |
| NCU00431T0 | NCU00431T0 | Neurospora crassa OR74A translocase of mitochondrial outer membrane 22 (155 aa) | 16787.95 | 154 | 9.090909091 | 1 | 1 | 1 |
| NCU00775T0 | NCU00775T0 | Neurospora crassa OR74A tricarboxylic acid-6 (386 aa) | 42033.64 | 385 | 2.337662338 | 1 | 1 | 1 |
| NCU00971T0 | NCU00971T0 | Neurospora crassa OR74A ribosomal protein S12 (153 aa) | 17353.4 | 152 | 8.552631579 | 1 | 1 | 1 |
| NCU01452T0 | NCU01452T0 | Neurospora crassa OR74A 40S ribosomal protein S1 (257 aa) | 29032.8 | 256 | 3.125 | 1 | 1 | 1 |
| NCU01689T0 | NCU01689T0 | Neurospora crassa OR74A mitochondrial DNA replication protein YHM2 (317 aa) | 34099.83 | 316 | 2.53164557 | 1 | 1 | 0 |
| NCU01689T1 | NCU01689T1 | Neurospora crassa OR74A mitochondrial DNA replication protein YHM2 (276 aa) | 29555.33 | 275 | 2.909090909 | 1 | 1 | 0 |
| NCU01826T0 | NCU01826T0 | Neurospora crassa OR74A DUF250 domain membrane protein (400 aa) | 43594.28 | 399 | 3.258145363 | 1 | 1 | 1 |
| NCU01949T0 | NCU01949T0 | Neurospora crassa OR74A unknown-16 (191 aa) | 21775.05 | 190 | 4.736842105 | 1 | 1 | 1 |
| NCU02260T0 | NCU02260T0 | Neurospora crassa OR74A regulatory particle, ATPase-like-3 (422 aa) | 46884.52 | 421 | 2.850356295 | 1 | 1 | 0 |
| NCU02295T0 | NCU02295T0 | Neurospora crassa OR74A mss4-like (1013 aa) | 111791.9 | 1012 | 1.28458498 | 1 | 1 | 1 |
| NCU02657T0 | NCU02657T0 | Neurospora crassa OR74A ethionine resistant-1 (396 aa) | 42941.11 | 395 | 3.037974684 | 1 | 1 | 0 |
| NCU02657T1 | NCU02657T1 | Neurospora crassa OR74A ethionine resistant-1 (396 aa) | 42941.11 | 395 | 3.037974684 | 1 | 1 | 0 |
| NCU02744T0 | NCU02744T0 | Neurospora crassa OR74A 60S ribosomal protein L9 (194 aa) | 21720.74 | 193 | 4.14507772 | 1 | 1 | 1 |
| NCU02905T0 | NCU02905T0 | Neurospora crassa OR74A 60S ribosomal protein L23 (140 aa) | 14671.88 | 139 | 5.755395683 | 1 | 1 | 1 |
| NCU03009T0 | NCU03009T0 | Neurospora crassa OR74A zuotin (446 aa) | 50564.06 | 445 | 1.573033708 | 1 | 1 | 0 |
| NCU03145T0 | NCU03145T0 | Neurospora crassa OR74A oxidative stress resistance (523 aa) | 56476.91 | 522 | 2.490421456 | 1 | 1 | 1 |
| NCU03565T0 | NCU03565T0 | Neurospora crassa OR74A ribosomal protein L26 (137 aa) | 15303.54 | 136 | 13.97058824 | 1 | 1 | 1 |
| NCU03800T0 | NCU03800T0 | Neurospora crassa OR74A endosomal cargo receptor (210 aa) | 23415.93 | 209 | 4.306220096 | 1 | 1 | 1 |
| NCU03953T0 | NCU03953T0 | Neurospora crassa OR74A NADH:ubiquinone oxidoreductase 19.3 (227 aa) | 24937.54 | 226 | 2.654867257 | 1 | 1 | 1 |
| NCU04013T0 | NCU04013T0 | Neurospora crassa OR74A yellow-1 (534 aa) | 59447.17 | 533 | 1.876172608 | 1 | 1 | 0 |
| NCU04013T1 | NCU04013T1 | Neurospora crassa OR74A yellow-1 (517 aa) | 57423.02 | 516 | 1.937984496 | 1 | 1 | 0 |
| NCU04114T0 | NCU04114T0 | Neurospora crassa OR74A hypothetical protein (72 aa) | 8220.058 | 71 | 18.30985915 | 1 | 1 | 1 |
| NCU04124T0 | NCU04124T0 | Neurospora crassa OR74A golgi membrane protein (744 aa) | 81205.05 | 743 | 1.480484522 | 1 | 1 | 1 |
| NCU04244T0 | NCU04244T0 | Neurospora crassa OR74A syntaxin-2 (336 aa) | 37282.71 | 335 | 2.089552239 | 1 | 1 | 1 |
| NCU04289T0 | NCU04289T0 | Neurospora crassa OR74A hypothetical protein (453 aa) | 50838.54 | 452 | 4.424778761 | 1 | 1 | 1 |
| NCU05137T0 | NCU05137T0 | Neurospora crassa OR74A non-anchored cell wall protein-1 (692 aa) | 75612.67 | 691 | 1.736613603 | 1 | 1 | 1 |
| NCU05234T0 | NCU05234T0 | Neurospora crassa OR74A GTP-binding protein ryh1 (214 aa) | 23375.81 | 213 | 5.164319249 | 1 | 1 | 1 |
| NCU05314T0 | NCU05314T0 | Neurospora crassa OR74A hypothetical protein (575 aa) | 62609.58 | 574 | 2.264808362 | 1 | 1 | 1 |
| NCU05363T0 | NCU05363T0 | Neurospora crassa OR74A 26S protease regulatory subunit 8 (390 aa) | 43539.05 | 389 | 3.084832905 | 1 | 1 | 0 |
| NCU05391T0 | NCU05391T0 | Neurospora crassa OR74A hypothetical protein (168 aa) | 18099.13 | 167 | 6.586826347 | 1 | 1 | 0 |
| NCU05454T0 | NCU05454T0 | Neurospora crassa OR74A glycerol-3-phosphate dehydrogenase (695 aa) | 76841.47 | 694 | 2.305475504 | 1 | 1 | 1 |
| NCU05526T0 | NCU05526T0 | Neurospora crassa OR74A lysine-5 (426 aa) | 46278.41 | 425 | 2.352941176 | 1 | 1 | 0 |
| NCU05526T1 | NCU05526T1 | Neurospora crassa OR74A lysine-5 (426 aa) | 46278.41 | 425 | 2.352941176 | 1 | 1 | 0 |
| NCU05526T2 | NCU05526T2 | Neurospora crassa OR74A lysine-5 (314 aa) | 34167.5 | 313 | 3.194888179 | 1 | 1 | 0 |
| NCU06361T0 | NCU06361T0 | Neurospora crassa OR74A cwl1 (330 aa) | 35704.58 | 329 | 3.647416413 | 1 | 1 | 0 |
| NCU06361T1 | NCU06361T1 | Neurospora crassa OR74A cwl1 (330 aa) | 35704.58 | 329 | 3.647416413 | 1 | 1 | 0 |
| NCU06361T2 | NCU06361T2 | Neurospora crassa OR74A cwl1 (319 aa) | 34643.07 | 318 | 3.773584906 | 1 | 1 | 0 |
| NCU06404T0 | NCU06404T0 | Neurospora crassa OR74A GTP-binding protein SAS1 (206 aa) | 22523.37 | 205 | 3.414634146 | 1 | 1 | 0 |
| NCU06454T0 | NCU06454T0 | Neurospora crassa OR74A cell division cycle 42-like (197 aa) | 21772.1 | 196 | 4.591836735 | 1 | 1 | 0 |
| NCU06454T1 | NCU06454T1 | Neurospora crassa OR74A cell division cycle 42-like (151 aa) | 16890.63 | 150 | 6 | 1 | 1 | 0 |
| NCU06508T0 | NCU06508T0 | Neurospora crassa OR74A compact (1123 aa) | 122402.4 | 1122 | 1.158645276 | 1 | 1 | 1 |
| NCU06854T0 | NCU06854T0 | Neurospora crassa OR74A RuvB-like helicase 2 (482 aa) | 52193.03 | 481 | 2.494802495 | 1 | 1 | 1 |
| NCU06871T0 | NCU06871T0 | Neurospora crassa OR74A doily (1956 aa) | 223261.3 | 1955 | 0.460358056 | 1 | 1 | 1 |
| NCU06882T0 | NCU06882T0 | Neurospora crassa OR74A RING-5 (538 aa) | 61831.38 | 537 | 1.117318436 | 1 | 1 | 0 |
| NCU06977T0 | NCU06977T0 | Neurospora crassa OR74A hypothetical protein (290 aa) | 33027.09 | 289 | 3.806228374 | 1 | 1 | 1 |
| NCU07075T0 | NCU07075T0 | Neurospora crassa OR74A calcium exchanger (508 aa) | 54373.12 | 507 | 2.366863905 | 1 | 1 | 0 |
| NCU07075T1 | NCU07075T1 | Neurospora crassa OR74A calcium exchanger (508 aa) | 54373.12 | 507 | 2.366863905 | 1 | 1 | 0 |
| NCU07367T0 | NCU07367T0 | Neurospora crassa OR74A regulatory particle, ATPase-like-4 (391 aa) | 43580.19 | 390 | 3.076923077 | 1 | 1 | 0 |
| NCU07461T0 | NCU07461T0 | Neurospora crassa OR74A DNA polymerase POL4 (847 aa) | 94524.9 | 846 | 0.709219858 | 1 | 1 | 1 |
| NCU07649T0 | NCU07649T0 | Neurospora crassa OR74A integral membrane protein (400 aa) | 42566.83 | 399 | 2.255639098 | 1 | 1 | 1 |
| NCU07697T0 | NCU07697T0 | Neurospora crassa OR74A tricarboxylic acid-4 (380 aa) | 40942.32 | 379 | 2.638522427 | 1 | 1 | 1 |
| NCU07721T0 | NCU07721T0 | Neurospora crassa OR74A regulatory particle, non-ATPase-like-1 (903 aa) | 100169.7 | 902 | 1.33037694 | 1 | 1 | 1 |
| NCU07723T0 | NCU07723T0 | Neurospora crassa OR74A norsolorinic acid reductase (381 aa) | 42341.15 | 380 | 1.842105263 | 1 | 1 | 0 |
| NCU07826T0 | NCU07826T0 | Neurospora crassa OR74A 40S ribosomal protein S19 (150 aa) | 16641.7 | 149 | 7.382550336 | 1 | 1 | 1 |
| NCU07849T0 | NCU07849T0 | Neurospora crassa OR74A thiamine-4 (539 aa) | 58376.55 | 538 | 1.486988848 | 1 | 1 | 1 |
| NCU07922T0 | NCU07922T0 | Neurospora crassa OR74A elongation factor 3 (1057 aa) | 117018.3 | 1056 | 1.041666667 | 1 | 1 | 1 |
| NCU08332T0 | NCU08332T0 | Neurospora crassa OR74A hexagonal-1 (177 aa) | 19096.62 | 176 | 6.25 | 1 | 1 | 1 |
| NCU08344T0 | NCU08344T0 | Neurospora crassa OR74A 60s ribosomal protein (123 aa) | 14038.59 | 122 | 7.37704918 | 1 | 1 | 1 |
| NCU08352T0 | NCU08352T0 | Neurospora crassa OR74A cysteine-9 (335 aa) | 35842.1 | 334 | 3.592814371 | 1 | 1 | 1 |
| NCU08380T0 | NCU08380T0 | Neurospora crassa OR74A plasma membrane phosphatase required for sodium stress response (449 aa) | 48435.11 | 448 | 2.232142857 | 1 | 1 | 0 |
| NCU08380T1 | NCU08380T1 | Neurospora crassa OR74A plasma membrane phosphatase required for sodium stress response (449 aa) | 48435.11 | 448 | 2.232142857 | 1 | 1 | 0 |
| NCU08380T2 | NCU08380T2 | Neurospora crassa OR74A plasma membrane phosphatase required for sodium stress response (397 aa) | 42973.51 | 396 | 2.525252525 | 1 | 1 | 0 |
| NCU08477T0 | NCU08477T0 | Neurospora crassa OR74A ypt-like-1 (204 aa) | 22444.14 | 203 | 3.448275862 | 1 | 1 | 0 |
| NCU08676T0 | NCU08676T0 | Neurospora crassa OR74A hypothetical protein (223 aa) | 23526.31 | 222 | 4.504504505 | 1 | 1 | 1 |
| NCU08693T0 | NCU08693T0 | Neurospora crassa OR74A heat shock protein 70-5 (669 aa) | 72669.02 | 668 | 1.047904192 | 1 | 1 | 0 |
| NCU08895T0 | NCU08895T0 | Neurospora crassa OR74A PNS1 (555 aa) | 60879.7 | 554 | 1.444043321 | 1 | 1 | 1 |
| NCU08897T0 | NCU08897T0 | Neurospora crassa OR74A protein transporter SEC61 subunit alpha (477 aa) | 52259.31 | 476 | 2.31092437 | 1 | 1 | 1 |
| NCU08980T0 | NCU08980T0 | Neurospora crassa OR74A NAD(P)H dehydrogenase (external)-2 (578 aa) | 64597.93 | 577 | 2.253032929 | 1 | 1 | 1 |
| NCU09476T0 | NCU09476T0 | Neurospora crassa OR74A 40S ribosomal protein S25 (98 aa) | 10772.95 | 97 | 10.30927835 | 1 | 1 | 1 |
| NCU09543T0 | NCU09543T0 | Neurospora crassa OR74A PQ loop repeat protein (286 aa) | 31733.16 | 285 | 3.50877193 | 1 | 1 | 1 |
| NCU11102T0 | NCU11102T0 | Neurospora crassa OR74A SCJ1 (427 aa) | 48257.62 | 426 | 3.521126761 | 1 | 1 | 1 |
