# Supplementary material for: Cellular Calcium Levels Influenced by NCA-2 Impact Circadian Period Determination in Neurospora
Source: mBio. 2021 Jun 29;12(3):e01493-21. doi: 10.1128/mBio.01493-21 (PMC8262947; doi:10.1128/mBio.01493-21)
Supplement: TABLE S2 [file mbio.01493-21-st002.docx]

**Table S2** Primers sets used in quantitative PCR

| **Primer name** | **Primer sequence** | **Target gene** |
| --- | --- | --- |
| *frq/qF* | TGG CTC GGA TAA GAA TGG TC | *frq* |
| *frq/qR* | ATG AAA GGT GTC CGA AGG TG |  |
| *wc-1/qF* | TCA GCA GCA TCA GTT CAA CC | *wc-1* |
| *wc-1/qR* | GTT GAT GTT CGC CCA TCT CT |  |
| *Cmk1/qF2* | ACGCCAAGGACTTCATTCTG | *camk-1* |
| *Cmk1/qR2* | TGGTAAGGTACGCTTTGATCTC |  |
| *Cmk2/qF* | CGGTTCATACTCGGTCGTTAAG | *camk-2* |
| *Cmk2/qR* | GAGCACGGCGATTTCATTG |  |
| *Cmk3/qF1* | AGATGGGCATTCATGGTTCG | *camk-3* |
| *Cmk3/qR1* | CTTCGGTAAGGAGCAGGTTG |  |
| *Cmk4/qF1* | CACGGGATTTGGAGGGTAAC | *camk-4* |
| *Cmk4/qR1* | TGTTGGGATGGTCAAGCTG |  |
| *rac1/qF* | GTC CAA GTG GCA CCC CGA GAT | *rac-1* |
| *rac1/qR* | TCC TTG GCG CAG TTG ACA CC |  |
